# Phase separation of Epstein-Barr virus EBNA2 protein reorganizes chromatin topology for epigenetic regulation

**DOI:** 10.1038/s42003-021-02501-7

**Published:** 2021-08-16

**Authors:** Yiting Yang, Xidong Ye, Ranran Dai, Zhaoqiang Li, Yan Zhang, Wei Xue, Yongchang Zhu, Delong Feng, Litao Qin, Xin Wang, Bo Lei, Shixiu Liao, Bingtao Hao

**Affiliations:** 1 Cancer Research Institute, School of Basic Medical Sciences, Southern Medical University, Guangzhou, China; 2 Henan Medical Genetics Institute, Henan Provincial Key Laboratory of Genetic Diseases and Functional Genomics, People’s Hospital of Zhengzhou University, Henan Provincial People’s Hospital, Zhengzhou, China; 3 Henan Eye Institute, Henan Eye Hospital, Zhengzhou University People’s Hospital, Henan Provincial People’s Hospital, Zhengzhou, China; 4 National Health Commission Key Laboratory of Birth Defects Prevention, Henan Key Laboratory of Population Defects Prevention, Zhengzhou, China

**Keywords:** Protein aggregation, Herpes virus, Virus-host interactions

## Abstract

Epstein-Barr virus nuclear antigen 2 (EBNA2) is a transactivator of viral and cellular gene expression, which plays a critical role in the Epstein-Barr virus-associated diseases. It was reported that EBNA2 regulates gene expression by reorganizing chromatin and manipulating epigenetics. Recent studies showed that liquid-liquid phase separation plays an essential role in epigenetic and transcriptional regulation. Here we show that EBNA2 reorganized chromatin topology to form accessible chromatin domains (ACDs) of the host genome by phase separation. The N-terminal region of EBNA2, which is necessary for phase separation, is sufficient to induce ACDs. The C-terminal domain of EBNA2 promotes the acetylation of accessible chromatin regions by recruiting histone acetylase p300 to ACDs. According to these observations, we proposed a model of EBNA2 reorganizing chromatin topology for its acetylation through phase separation to explain the mechanism of EBNA2 hijacking the host genome by controlling its epigenetics.

## Abstract (web summary)

Yang et al find that phase separation of the Epstein-Barr virus nuclear antigen 2 (EBNA2) is involved in the formation of accessible chromatin domains of the host genome. They also find that EBNA2 recruits histone acetyltransferase to promote histone acetylation on accessible chromatin regions and regulate gene expression and that these two functions are performed by the N- and Cterminus respectively.

## Introduction

The Epstein-Barr virus, a member of the human herpesvirus family, is one of the most common human viruses^1,2^. It has been reported to be associated with many human diseases, including infectious mononucleosis, lymphoma, gastric cancer, nasopharyngeal, dermatomyositis, systemic lupus erythematosus, rheumatoid arthritis, and multiple sclerosis^1^. EBNA2 is the main transactivator of the virus nuclear proteins and regulates latent viral transcription and genes of host cells^3^. EBNA2 also plays an essential role in EBV-mediated B cell immortalization^4,5^. A recent study showed that EBNA2 and its associated transcription factors and cofactors occupy a substantial fraction of autoimmune risk loci^6^, indicating its role in the EBV-associating autoimmune diseases.

Liquid−liquid phase separation (LLPS) is a process in which macromolecular spontaneously separates into two phases, one phase with highly concentrated macromolecular^7,8^. It has been reported that phase separation drives the assembly of membraneless compartments in cells, including stress granule^9–11^, Cajal bodies, and nucleoli. Several chromatin-related proteins like HP1^12,13^ and BRD4^14^ have the potential of forming liquid droplets, indicating the role of LLPS in epigenetic regulation^15^. Recent two studies showed that chromatin undergoes LLPS under physiologic conditions^16^. Many transcription factors contain activation domains that have an intrinsic feature of LLPS. It was proposed that transcription factors recruit co-activators and RNA polymerase pol II through the phase-separation capacity of their activation domains to form enhancers and super-enhancers and regulate gene transcription.

EBNA2 interacts with histone acetyltransferases (HATs), transcription factors, and the basal transcription machinery to control host gene expression^17–22^. It has been reported that EBNA2 regulates MYC and RUNX3 expression by hijacking super-enhancers characterized by LLPS^23^. Early observation showed that EBNA2 protein forms granules in the nucleoplasm, the chromatin fraction, and the nuclear matrix, suggesting its potential of LLPS^24^. A recent study reported that EBNA2 has an intrinsic feature of phase separation and form nuclear puncta with a property of liquid-like condensates^25^. But how phase separation of EBNA2 regulates chromatin topology for epigenetic regulation is unknown.

Here we analyzed the key domain of EBNA2 for phase separation and its role in chromatin topology using an assay of transposase-accessible chromatin with visualization (ATAC-see). We found that EBNA2 induced accessible chromatin domains (ACDs), which were colocalized with and depended on EBNA2 condensates. The C-terminal transactivation domain (C-TAD) of EBNA2 recruits histone acetyltransferase p300 into ACDs to acetylate histone H3K27 in the accessible chromatin regions. We report these findings as follows.

## Results

### EBNA2 forms liquid-like puncta in the nucleus

**Fig. 1:**
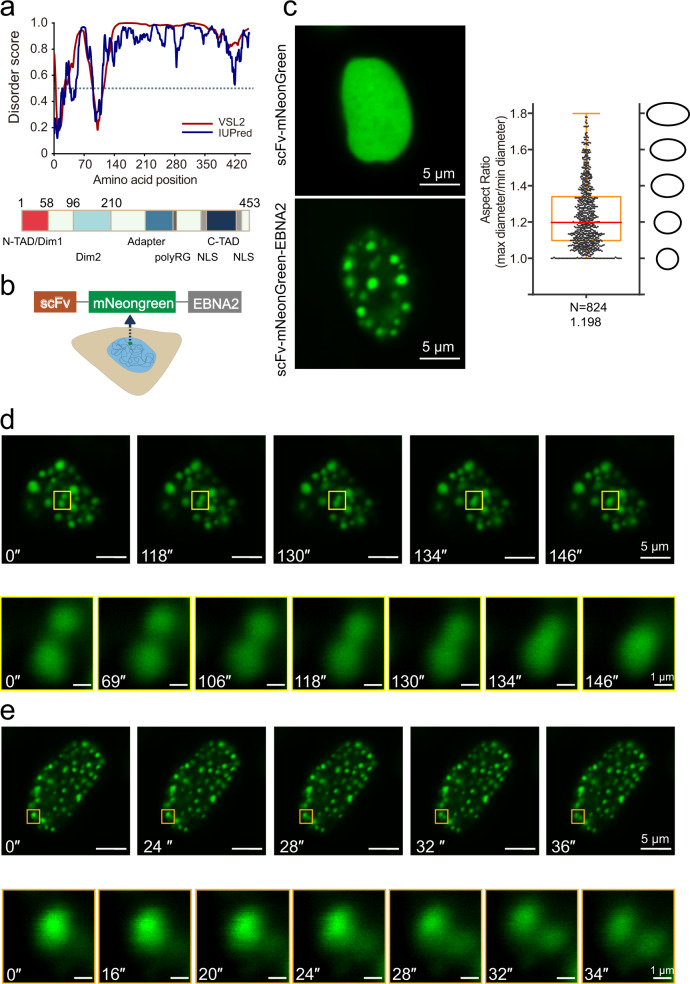
EBNA2 forms liquid-like puncta in the nucleus. **a** Disorder sequence analysis of EBNA2 protein (top) using the algorithms IUPred (blue) and VSL2 (magenta). IUPred and VSL2 scores are shown on the *y* axis, and amino acid positions are shown on the *x-*axis. The dotted line indicates 0.5 disordered score. The diagram below shows the domains and motifs of EBNA2 protein: N-TAD N-terminal transactivation domain; C-TAD C-terminal transactivation domain; Dim1 and Dim2 dimerization motif 1 and 2; NLS nuclear localization signal; and adapter region. **b** Schematic representation for the scFv- mNeonGreen-EBNA2 plasmid. **c** Live imaging of HEK 293T cells transfected with scFv- mNeonGreen-EBNA2 and scFv mNeongreen plasmids (left). Box plots showing the distribution of aspect ratios for droplets of mNeonGreen-EBNA2 (right). The numbers of droplets examined and the mean aspect ratios are shown. Box plot represents min to max. **d**, **e** Time-lapse images of the nucleus of a HEK 293T cell transiently transfected scFv-mNeongreen-EBNA2 subjected to laser excitation every 4 s for the times indicated. A droplet fusion and fission event occur respectively in the region highlighted by the yellow (**d**) and orange box (**e**).

The EBNA2 containing 453 amino acids generated from the laboratory EBV strain HONE-1 (type1) was used in this study (Fig. S1a)^26^. Sequence analysis of EBNA2 protein using two IDR prediction programs, VSL2 and IUPred, revealed that EBNA2 has an extended C-terminal disorder region, containing an adapter and a transcription activation domain (C-TAD) (Fig. 1a). We also analyzed the EBNA2 sequences from EBV-1 strain B95-8 and EBV-2 strain AG876 (Fig. S1b). The B95-8 EBNA2 has a broader N terminal IDR due to the longer polyproline region in the N terminal. Although the amino acid sequence of the AG876 EBNA2 is different from that of type 1 EBNA2, IDR regions and scores are similar (Fig. S1b).

EBNA2 was constructed in the expressing plasmid of scFv-mNeonGreen, and CTCF-IDR was used as a control (Fig. 1b and S2)^27^. EBNA2 but not CTCF-IDR formed puncta in the nucleus of the transfected HEK 293T cells and EBV positive Raji cells (Fig. 1c and S2, S3), which is consistent with the previous report^24^. The analysis of aspect ratio demonstrated that the EBNA2 droplets tended to a spherical shape, and the median aspect ratio (1.2) was close to 1 (Fig. 1c). Live cell imaging showed that the EBNA2 droplets undergo fusion and fission (Fig. 1d, e). These results indicated that EBNA2 puncta exhibited characteristics of liquid-like droplets.

**Fig. 2:**
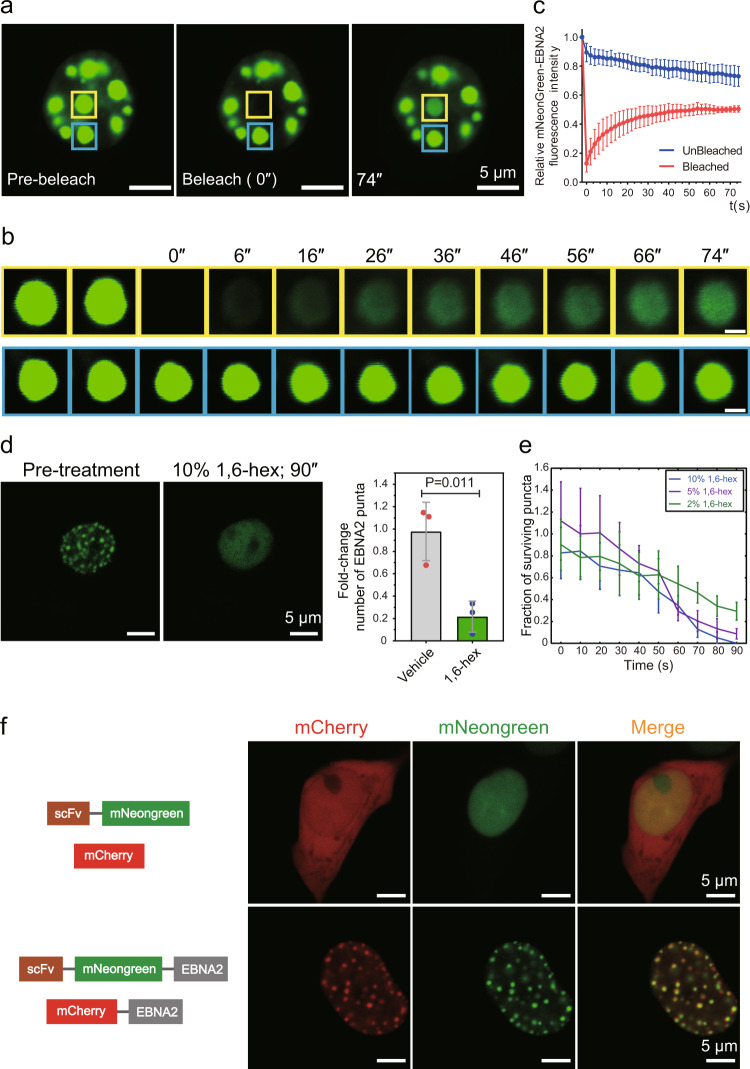
EBNA2 undergoes phase separation in host cells. **a** Image and **b** time-lapse and close-up view of a mNeonGreen EBNA2 droplet (yellow box) before (left), during (middle), and after (right) photobleaching. The blue box highlights an unbleached region for comparison. Time relative to photobleaching (0 s) indicated. Scale bars, 1 μm (**b**). **c** Quantification of FRAP for mNeonGreen-EBNA2 puncta. The bleaching event occurs at *t* = 0 s. For the bleached area and the unbleached control, background-subtracted fluorescence intensities are plotted relative to a prebleach time point (*t* = −2 s). Data are plotted as mean + SD (*n* = 3). **d** Representative images of mNeonGreen-EBNA2 before and after treatment with 10% 1,6-hexanediol for 90 s (left). The fold change of the number of mNeonGreen-EBNA2 puncta observed after the addition of 1,6-hexanediol to the final concentration of 10% (right). **e** Number of nuclear puncta formed by mNeonGreen-EBNA2 surviving over time upon addition of 1,6-hexanediol at different concentrations. Error bars represent SE. **f** Schematic representation of recombinant mNeonGreen/mCherry fusion proteins used in this experiment (left). The mNeonGreen-EBNA2, mCherry-EBNA2, mNeonGreen, and mCherry plasmids were transiently transfected into 293T cells. Double immunofluorescence revealed co-localization of mNeonGreen-EBNA2 and mCherry-EBNA2 proteins in HEK 293T cells (right).

To further explore the dynamic liquid-like feature of the EBNA2 puncta, we performed the fluorescence recovery after photobleaching (FRAP) experiment. After photobleaching, EBNA2 puncta recovered fluorescence on a time scale of minutes (Fig. 2a–c). 1,6-hexanediol is a widely used tool to probe LLPS in cells^28^. After adding 1,6-hexanediol to HEK 293T cells transfected with EBNA2-expressing plasmid for 90 s, EBNA2 droplets were significantly dispersed (Fig. 2d, e, Movies S1−3). The expression of EBNA2 in HEK 293T cells was slightly lower than that in Raji cells (Fig. S4a), indicating that phase separation was not caused by overexpression. We also made a mCherry-ENBA2 expressing construct (Fig. S5). The mCherry-EBNA2 did not form puncta in the nucleus after transfected in HEK293T cells (Fig. S5). We compared mCherry-ENBA2 and mNeonGreen-EBNA2 mRNA expressions using reverse-transcription quantitative PCR and observed that mCherry-EBNA2 was three folds lower than mNeonGreen-EBNA2 (Fig. S4b), which may explain the inability of mCherry-ENBA2 to form puncta. However, we observed the mCherry-EBNA2 puncta in HEK 293T cells co-transfected with mCherry-EBNA2 and mNeonGreen-EBNA2 constructs (Fig. 2f). There is a strong self-association of ENBA2, and most of the mCherry signals were overlapped with the mNeonGreen signals (Fig. 2f). These results indicated that EBNA2 undergoes LLPS in the nucleus.

### The N terminal of EBNA2 contributes to the phase separation

**Fig. 3:**
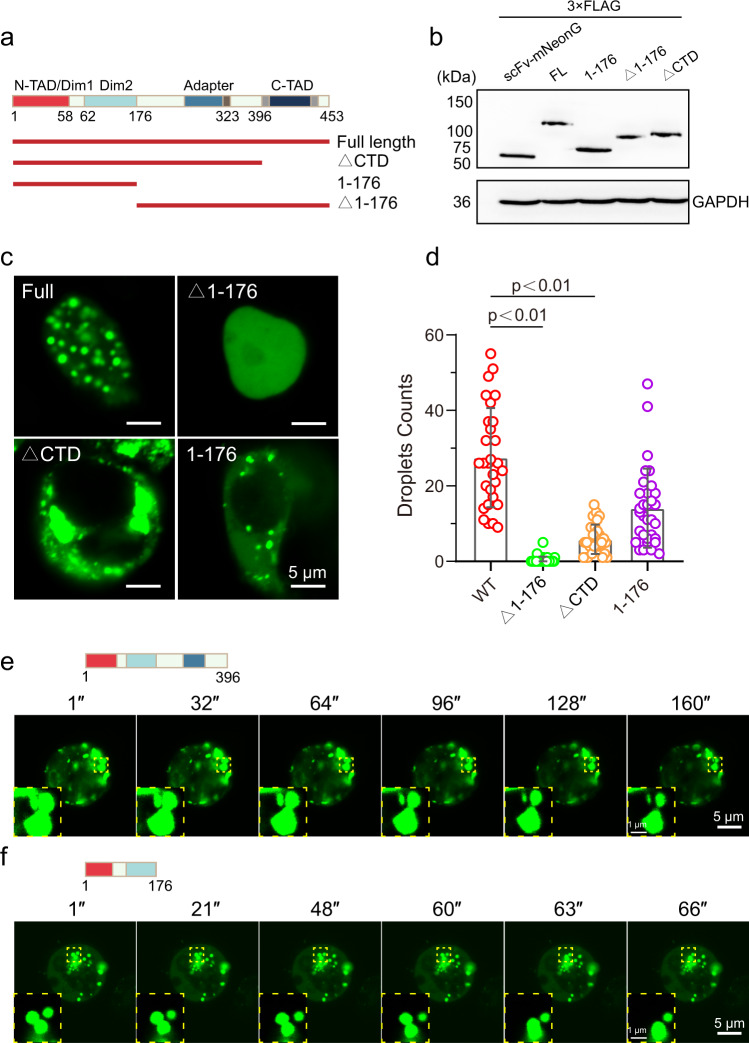
The N-terminal of EBNA2 is necessary for phase separation. **a** Diagram of the Full length and truncated EBNA2. **b** Western blot of scFv-mNeonGreen protein, and full length and truncates of scFv-mNeonGreen EBNA2 protein. **c** Immunofluorescence images of the truncated EBNA2 in HEK 293 T cells (left). **d** The droplet counts per area of the mNeonGreen-truncated-EBNA2. Error bars represent SD. **e**, **f** Time-lapse images of both mNeonGreen-EBNA2(1-396) and mNeonGreen-EBNA2(1-176). Zoom in shows two truncated EBNA2 fusion events. Scale bars, 5 μm, and zoom in 1 μm.

EBNA2 protein contains several domains: Two N-terminal dimerization domains, Dim1 and Dim2, separated by a poly-proline stretch (poly-P), the middle adapter mediating promoter targeting by association with CBF1, the RG repeat (polyRG) a modulator of EBNA2 activity^29^, and the C-terminal transactivation domain (C-TAD). The disorder region of EBNA2 is located at the region containing part of the Dim2, the adapter, and the C-TAD. To explore the region that contributes to the phase separation of EBNA2, we made six truncates of EBNA2: ΔCTD (deletion of 397-453), Nter (1-176), Cter (177-453), ΔpolyRG (deletion of 311-322), ΔpolyRG + CTD (324-453), ΔpolyRG + ΔCTD (deletion of 310-453), and a mutant W424T which disrupts EBNA2-p300 association^30^ (Fig. 3a, b and S6). To our surprise, the ΔCTD and Nter truncated EBNA2 still formed liquid-like puncta in the cytoplasm, although they cannot enter the nucleus due to a lack of nuclear localization signals (NLS) (Fig. 3c–f). Deletion of polyRG or mutation W424T did not affect the formation of EBNA2 condensates (Fig. S6). While the C-terminal EBNA2 almost lost the ability to form puncta, although it contains most of the IDR (Fig. 3c). Both Dim1 and Dim2 domains mediate the homotypic adhesion of EBNA2, indicating the critical role of self-association in EBNA2 phase separation.

### EBNA2 induced and co-localized spatially segregated ACDs

**Fig. 4:**
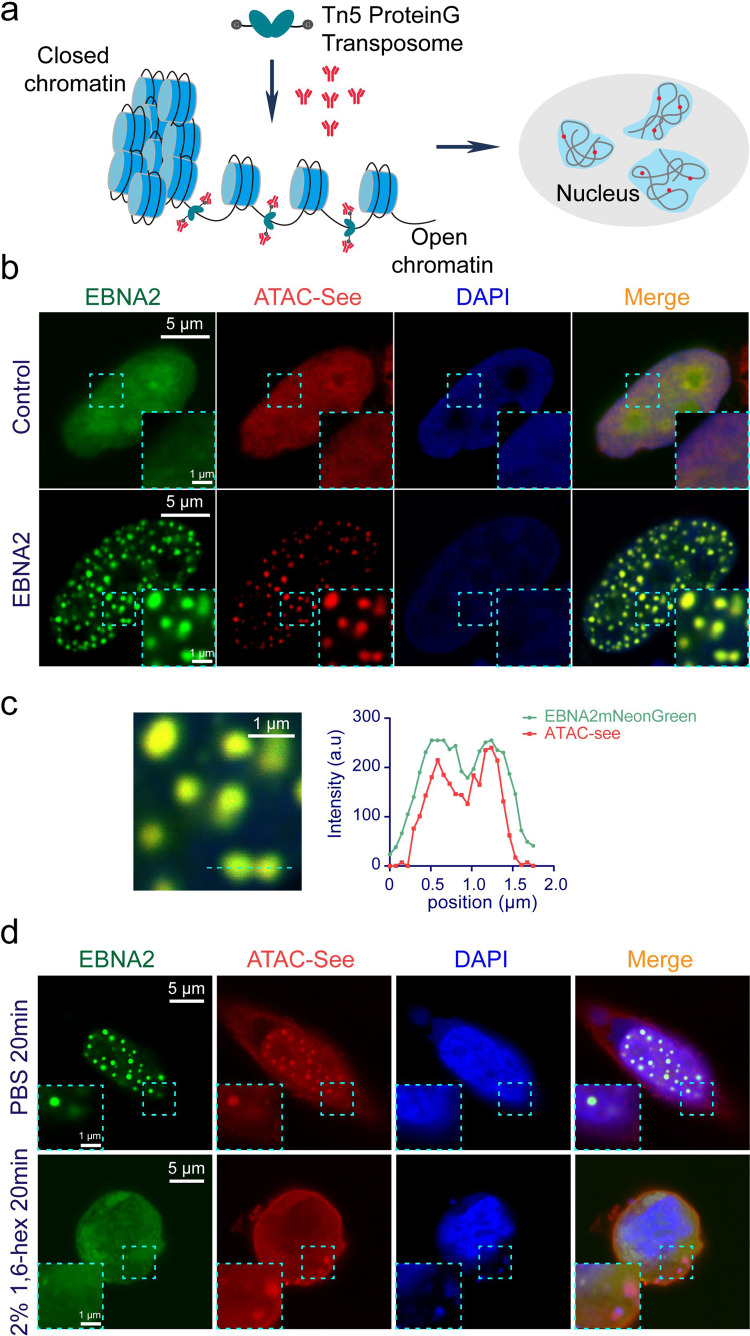
EBNA2 reorganized chromatin topology to form accessible chromatin domains. **a** The modified ATAC-see schematic diagram. The protein G-fused Tn5 adapter transposase (pG-Tn5) forms the active transposome complex in vitro. The cells were fixed, permeabilized, and the accessible sites were labeled with the pG-Tn5 transposome. The pG-Tn5 was stained with rabbit-derived IgG DyLight^TM^ 549 conjugated antibody. **b** Representative images show the colocalization of the accessible chromatin domains and mNeonGreen-EBNA2 condensates. The experiment was repeated three times independently. Scale bars, 5 μm. Magnified regions, scale bars, 1 μm. **c** Magnification of the box region (left) in (**b**) and line plot of the dotted line in the magnified image (right). Scale bars, 1 μm. **d** Representative images show the colocalization of the accessible chromatin domains and EBNA2 condensates after treatment with PBS or 2% 1,6-hex for 20 min. The experiment was repeated three times independently. Scale bars, 5 μm. Magnified region scale bars, 1 μm.

Eukaryotic genomes are extensively compacted in chromatin, while active regulatory elements are accessible for binding by transcription factors. Recent studies showed active enhancers cluster with transcription factors and cofactors to form super-enhancers, which has been proved to have features of LLPS^14^. By using ATAC (assay of transposase-accessible chromatin) based visualization techniques including ATAC-see or ATAC-PALM, researchers found that accessible chromatin regions cluster to form spatially segregated ACDs^31,32^. It was reported that hyperosmotic treatment with sorbitol induced large ATAC-labeled clusters^33^. EBNA2 can incorporate into super-enhancers in B cells to regulate B cell-specific gene expression^23^. We asked whether EBNA2 condensates co-localize with the ACDs. We modified the ATAC-see assay by using Protein G-fused Tn5 transposome instead of Tn5 transposase coupled with fluorescence-conjugated DNA adaptors, and then detecting the Tn5 with fluorescence-labeled antibody (Fig. 4a).

ATAC-see imaging revealed that accessible chromatin regions were widely dispersed throughout the nucleus and distinct from the DAPI signal (tightly compacted DNA) in the HEK293T cells (Fig. 4b). To our surprise, EBNA2 dramatically changed the ATAC-labeled signal distribution and formed large ACDs across the nucleus (Fig. 4b). Moreover, all ATAC-labeled structures were associated with the mNeonGreen-EBNA2 condensates (Fig. 4b, c). We also detected the EBNA2 variant derived from the B95-8 EBV strain, and the result showed that both the B95-8 and HONE-1 EBNA2 induced ACDs in BJAB cells, an EBV negative Human Burkitt lymphoma B cell (Fig. S7). To test whether the EBNA2-induced ACDs were dependent on phase separation, we treated cells with 1,6-hexanediol. The EBNA2 puncta and most ATAC clusters disappeared after 20 min of 1,6-hexanediol treatment (Fig. 4d). The result suggested that the phase separation of EBNA2 reorganizes chromatin topology to form spatially segregated ACDs in the nucleus.

### The N-terminal of EBNA2 is sufficient to induce ACDs

**Fig. 5:**
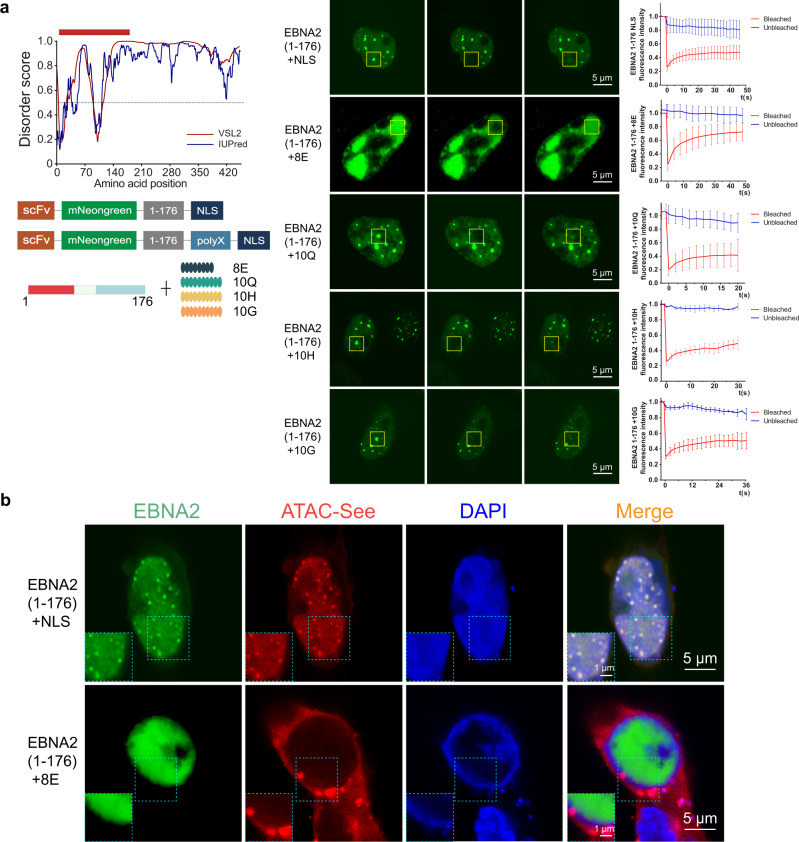
The N-terminal of EBNA2 is necessary and sufficient to induce ACDs. **a** Immunofluorescence images of the polyX-modified EBNA2 N-terminal. Graphs plotting intrinsic disorder analysis for EBNA2 and the design of the polyX-modified EBNA2 N-terminal (left). The sequence (amino acid from 1 to 176) cloned for subsequent experiments is highlighted with a red bar. Below is a schematic diagram of the polyX-modified EBNA2 N-terminal. FRAP recovery images of the EBNA2(1-176), EBNA2(1-176) + 8E, EBNA2(1-176) + 10Q, EBNA2(1-176) + 10H, and EBNA2(1-176) + 10G condensates (middle). The yellow box highlights the condensate before, during, and after photobleaching. Data are plotted (right) as SD (*n* = 3). Scale bars, 5 μm. **b** Representative images show the colocalization of the accessible chromatin domains and mNeonGreen-EBNA2(1-176) (top) or mNeonGreen-EBNA2(1-176) + 8E (bottom) condensates. The experiment was repeated three times independently with similar results. Scale bars, 5 μm. Magnified regions, scale bars, 1 μm.

The above results showed that the N terminal of EBNA2 is necessary and sufficient for phase separation of EBNA2. To investigate the role of EBNA2 phase separation in the formation of ACDs, we added a nuclear localization signal to the C terminal of the EBNA2 Nter truncate and made a construct expressing mNeonGreen-Nter-NLS protein. After transfected in HEK 293T cells, the mNeonGreen-Nter-NLS formed puncta in the nucleus (Fig. 5a). The FRAP result showed the liquid-like droplets of the Nter(1-176) in the nucleus (Fig. 5a). Then we did the ATAC-see imaging in the HEK 293T cells transfected with the mNeonGreen-Nter-NLS plasmid. We observed the formation of spatially segregated ACDs in the nucleus, and the Nter droplets were overlapped with all ACDs (Fig. 5b). It suggested that the N-terminal region of EBNA2 is sufficient to induce ACDs in the nucleus.

To explore the role of the phase separation of the EBNA2 in inducing ACDs, we made four designs of adding polyX amino acids at the tail of the Nter truncate to interfere with phase separation (Fig. 5a). We found that the Nter truncate condensates were not disrupted in three designs of the Nter plus ten glutamines (Nter-10Q), Nter plus ten histidines (Nter-10H), and Nter plus glycines (Nter-10G) (Fig. 5a). However, the Nter plus eight glutamic acids (Nter-8E) changed the distribution of the Nter truncate in the nucleus, and the mNeonGreen-Nter-8E formed a large plague instead of droplets (Fig. 5a). The FRAP experiments showed that the Nter-8E is highly dynamic in a large plague (Fig. 5a), indicating that the negatively charged poly-E interfered with the phase separation. The ATAC-see assay showed that the Nter-8E did not induce ACDs in the nucleus (Fig. 5b). The results suggested that the phase separation of EBNA2 is necessary for inducing ACDs.

### EBNA2 recruits histone acetyltransferase p300 for histone H3K27 acetylation

**Fig. 6:**
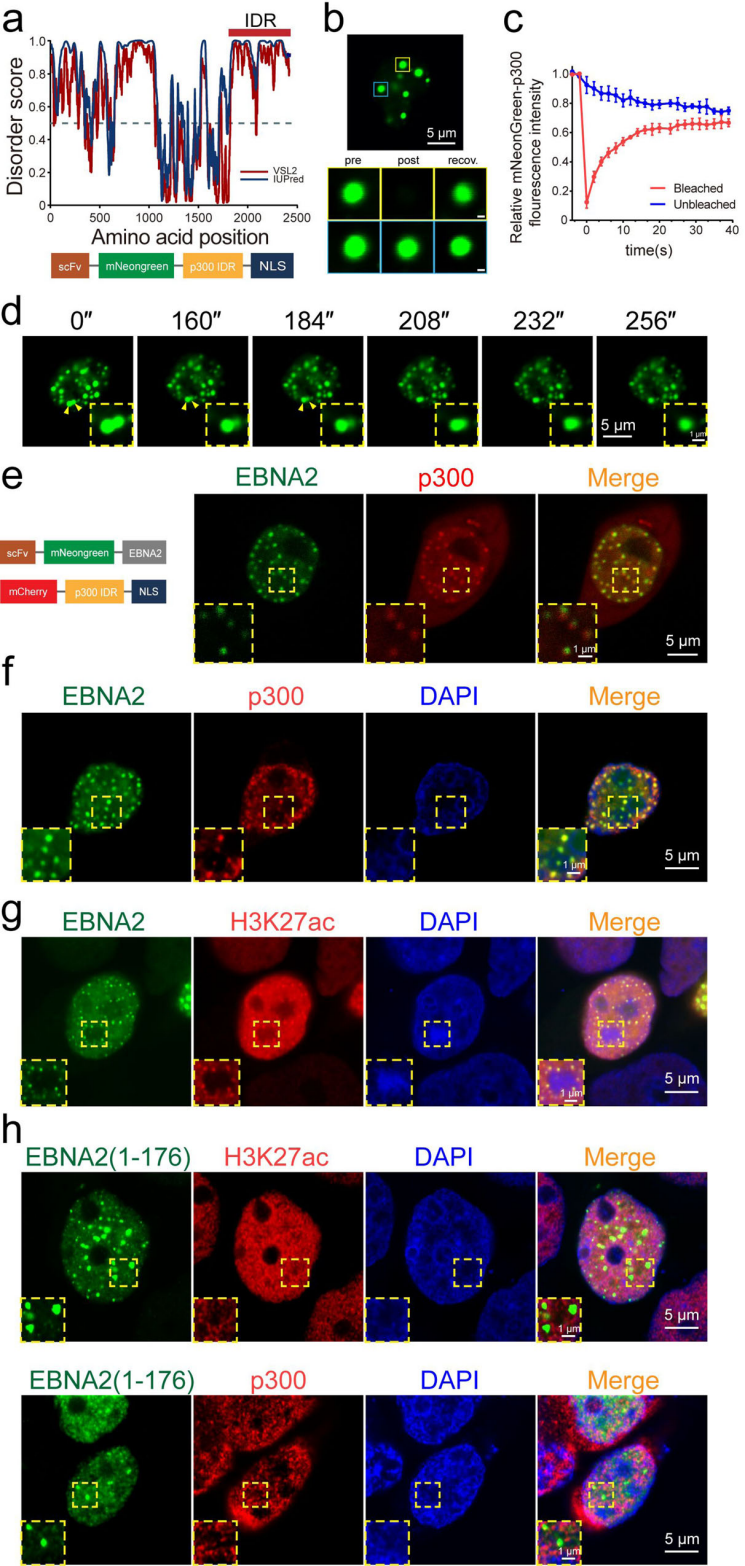
EBNA2 recruits p300 for histone H3K27 acetylation. **a** Disorder analysis of the histone acetyltransferase p300 2415 amino acid (top). The algorithms used were: IUPred (blue) and VSL2 (magenta). The IDR (amino acid from 1851 to 2415) cloned for subsequent experiments is highlighted with a red bar. The schematic below representation of the p300 IDR plasmid. The plasmid consists of the p300 IDR fused to scFv-mNeongreen and NLS. **b** Live cell images of HEK 293T cells transfected with mNeonGreen-p300-IDR plasmid (top) and a mNeonGreen-p300-IDR droplet (bottom, yellow box) before (left), during (middle), and after (right) photobleaching. The blue box highlights an unbleached region for comparison. Scale bars, 1 μm. **c** Quantification of FRAP data for mNeonGreen-p300-IDR droplets. The bleaching event occurs at *t* = 0 s. For the bleached area and the unbleached control, background-subtracted fluorescence intensities are plotted relative to a prebleach time point (*t* = −4 s). Data are plotted as mean + SD (*n* = 3). **d** Example of p300-IDR fusion (yellow arrowheads and magnified regions), scale bars, 5 μm. Magnified regions, scale bars, 1 μm. **e** Double immunofluorescence imaging revealed co-localization of EBNA2 and p300 IDR in HEK 293T cells. Insets are enlarged areas indicated in the main panel. Scale bars, 5 μm. Magnified region scale bars, 1 μm. **f** Colocalization between stably expressing EBNA2 and endogenous p300 by Immunofluorescence in HEK 293T cells. Double immunofluorescent staining revealed co-localization of EBNA2 and p300. Insets are enlarged areas indicated in the main panel. The experiment was repeated three times independently with similar results. Scale bars, 50 μm. Magnified regions, scale bars, 5 μm. **g** Representative immunofluorescence images showing colocalization of mNeonGreen-EBNA2 condensates with H3K27ac foci in HEK 293T cells. The experiment was repeated three times independently with similar results. Scale bars, 5 μm. Magnified regions, scale bars, 1 μm. **h** Representative immunofluorescence images of HEK 293T cells transfected with mNeonGreen-EBNA2(1-176). The p300 and H3K27ac were stained with the specific antibodies. The experiment was repeated three times independently with similar results. Scale bars, 5 μm (**b**, **d**, **e**, **g**, **h** (whole-cell images)), 1 μm (**b**, **d**, **e**, **g**, **h** (magnified views of the boxed regions)).

It was reported that EBNA2 interacts with p300, CBP, and PCAF histone acetyltransferases^22^. We analyzed the disorder region of p300 and found that the p300 C-terminal contains a strong IDR (Fig. 6a). We generated a construct of mNeonGreen-p300IDR and transfected HEK 293T cells. The mNeonGreen-p300IDR formed puncta in the nucleus and cytoplasm (Fig. 6b). Rapid FRAP and droplet fusion showed that p300IDR had phase separation characteristics (Fig. 6b–d). Most of the p300IDR droplets were associated with the EBNA2 condensates in the nucleus after the co-transfection of mNeonGreen-EBNA2 and mCherry-p300IDR constructs (Fig. 6e). We verified the association of the endogenous p300 puncta with the EBNA2 condensates (Fig. 6f and S8a). CBP/p300 acetylates several specific lysine residues of histone, including H3K4, H3K9, and H3K27^34^. H3K27 acetylation (H3K27ac) marks active enhancers and promoters. We observed that all H3K27ac spots were associated with the mNeonGreen-EBNA2 condensates (Fig. 6g and S8b).

EBNA2 has two transactivation domains: Dim1 as N-terminal transactivation domain (N-TAD) and C-terminal transactivation domain (C-TAD). It was reported that both transactivation domains can recruit histone acetyltransferase activity by interacting with CBP, p300, and PCAF^22^. However, we observed the reduced association between the EBNA2 N-terminal (1-176) and p300 (Fig. 6h and S9a). The association of the EBNA2 N-terminal with H3K27ac spots also decreased dramatically (Fig. 6h and S9b). The result indicated that the EBNA2 C-TAD plays a critical role in recruiting p300 to EBNA2 condensates.

### EBNA2 promotes the acetylation of histone H3K27 in genome-wide

**Fig. 7:**
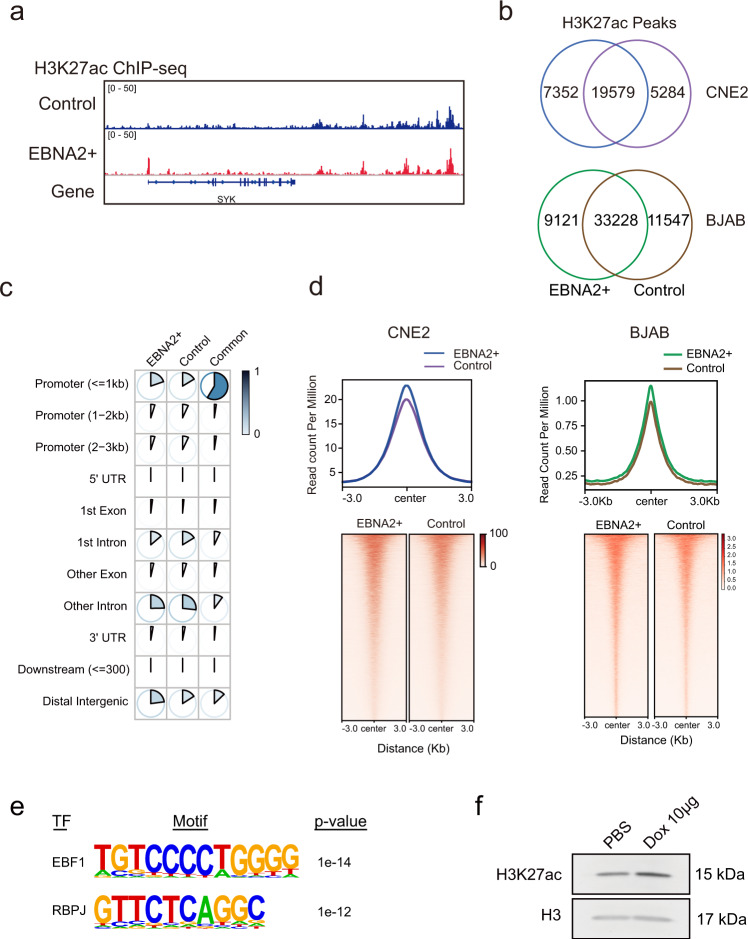
ENBA2 promoted the acetylation of the histone H3K27 in genome-wide. **a** ChIP-seq tracks of H3K27ac in the Dox-induced EBNA2+ CNE2 cells (blue track) and control CNE2 cells (purple track). **b** Venn diagram of H3K27ac peaks in the Dox-induced EBNA2+ CNE2 cells (green track) and control CNE2 cells (brown track), EBNA2+ BJAB cells (green track), and control cells (brown track) **c** Annotation of H3K27ac peaks. EBNA2+: EBNA2+ cell unique peaks, control: control cell unique peaks, common: peaks of overlapped in both cell types. **d** The plot and heatmap of common peaks in the Dox-induced EBNA2+ cells (blue track) and control cells (purple track), EBNA2+ BJAB cells (green track), and control cells (green track). **e** Selected transcription factor binding motifs overrepresented in CUT&Tag peaks, the most enriched factor for each family is shown. **f** Western blot of H3K27ac of the EBNA2-expressing HEK 293T cells treated with 10 μg/mL Doxycycline or vehicle for 24 h. H3 served as a loading control.

To explore the role of the EBNA2 in promoting histone H3K27 acetylation in genome-wide, we did H3K27ac ChIP-seq in human nasopharyngeal carcinoma cell line CNE2 cells with DOX-induced EBNA2 and control cells treated with DOX (Fig. S10). After mapping, the H3K27ac signals were visualized in the genomic data visualization tool IGV (Integrative Genomics Viewer)^35^. We observed an increased H3K27ac signal on the promoter of the *Syk*, a known EBNA2 target gene, in the EBNA2+ cells (Fig. 7a). After peak calling, we got 26731 H3K27ac peaks from the EBNA2+ cells, of which 7352 peaks were unique in the EBNA2+ cells (Fig. 7b). We annotated the common and unique peaks and found that about 60% of the common peaks were located at promoter regions (Fig. 7c). In contrast, the unique peaks in the promoter regions were much less (Fig. 7c). More unique peaks were found in introns and intergenic regions (Fig. 7c), indicating the role of EBNA2 in regulating enhancers. Analysis of the signals of the common peaks showed that the H3K27 acetylation level at the common peaks was higher in the EBNA2+ cells (Fig. 7d), consistent with the observation of evaluated H3K27ac in the whole cells (Fig. 7f). The results suggested that EBNA2 promoted histone H3K27 acetylation in genome-wide. Meanwhile, we also detected genome-wide H3K27ac modification in BJAB cells with and without transiently transfected EBNA2 using CUT&Tag sequencing, a recently developed technique for analyzing protein-DNA interactions with a small number of cells (Fig. 7). The sorted mNeonGreen-positive cells were treated with anti-H3K27ac antibody and protein G-Tn5 transposase after permeabilization. The libraries were amplified for deep-sequencing on the Illumina sequencing platform. After mapping, we got 42349 H3K27ac peaks from the EBNA2+ cells, of which 9121 peaks were unique in the EBNA2+ cells (Fig. 7b). Analysis of the signal strength of the common peaks showed that the H3K27 acetylation level of the common peaks was higher in the EBNA2+ cells (Fig. 7d), suggesting that EBNA2 promoted histone H3K27 acetylation genome-wide the same as in CNE2 cells. We analyzed the motifs of the unique peaks in EBNA2 expressing cells and found that the binding motifs of the transcription factors EBF1 and RBPJ were enriched in the EBNA2 specific peaks (Fig. 7e). Previous studies have shown that EBNA2 activates transcription through interaction with the transcription factor RBPJ in lymphoblastoid cell lines (LCLs)^36^. EBNA2 also forms complexes with early B cell factor 1 (EBF1), a B cell-specific DNA binding transcription factor, and EBF1 stabilizes EBNA2 chromatin binding^37^. The result indicated that EBNA2 promotes the H3K27 acetylation of the RBPJ and EBF1 binding regions in B cells.

The ATAC-see result showed that EBNA2 promoted open chromatin condensation, which might change chromatin accessibility and conformations genome-wide. To detect the chromatin accessibility changes in the whole genome, we performed an ATAC-seq experiment in BJAB cells with and without transiently transfected EBNA2. We observed a slight increase of ATAC peak number and a slight decrease of chromatin accessibility of the common peaks, indicating that EBNA2 has few effects on chromatin accessibility (Fig. S11). Next, to detect the chromatin conformation changes in the whole genome, we performed a Hi-C experiment. The results showed that the strength of chromatin loops in HEK 293T cells transfected with EBNA2 N-terminal expressing plasmid increased, and the number of chromatin loops increased throughout the genome (Fig. S12). The result suggested that EBNA2 promotes genome-wide chromatin interactions.

## Discussion

Evidence is now mounting that LLPS is involved in a wide range of biological processes^7,8,38^, including the formation of membraneless bodies such as nucleoli^39,40^, assembly of stress granules^9,10,28^, membrane receptor clusters at the cell membrane^14,41–44^, transcription regulation, and chromatin dynamics^13,15,16,45^. It was reported that activation domains of transcription factors form phase-separation condensates with the Mediator coactivator to activate genes^46^. Denes Hnisz et al. proposed a phase separation model of enhancer and super-enhancer assembly and function for transcription regulation and elongation^47^. EBNA2 not only activates viral genes but also universally regulates host gene expression through epigenetic regulation^3^. It has been reported that EBNA2 regulates host gene expression by hijacking the super-enhancers of the host cell^23,48,49^. But the mechanism is unknown. Here, we propose a model in which phase separation of EBNA2 reorganizes chromatin topology to form ACDs in EBNA2 condensates and recruits histone acetyltransferase p300 for histone H3K27 acetylation in the accessible regions. The model may explain the observation that the EBNA2 binding sites were in broad clusters to form super-enhancers and mediated the interactions between enhancers/super-enhancers and promoters in the host genome^23,48,49^.

ATAC-see is a recently developed assay for a transposase-accessible-chromatin-based imaging method to visualize accessible chromatin^31^. Researchers observed that the ATAC-see signals were concentrated into several nuclear foci in some cells^31^, suggesting that the accessible genome regions form a higher structure in the nucleus. Xie et al. showed that the spatially segregated ACDs were associated with active chromatin and transcribed genes^32^. They also found that acute CTCF depletion markedly enhanced accessible chromatin clustering^32^. A recent study showed that large ATAC-labeled clusters were quickly induced after the hyperosmotic reagent D-sorbitol treatment, and the ACDs were overlapped with the nuclear condensates of Yes-associated protein (YAP), a mechano-chemical stress-response protein in the Hippo pathway^33^. Here, we found that phase separation of EBNA2 is necessary to induce ACDs in the nucleus, and 1,6-hexanediol treatment disrupted both EBNA2 condensates and ACDs. Recently studies demonstrated the ability of chromatin to undergo LLPS^13,16,45^. These facts imply that the ACDs have the characteristics of LLPS. Due to the lack of suitable markers for ACDs, we cannot verify the phase separation characteristics of the ACDs in living cells.

Gibson et al. demonstrated histone modification is critical for chromatin phase separation, in which histone acetylation by p300 antagonizes phase separation^45^. But multi-bromodomain proteins, such as BRD4, drive acetylated chromatin to form a new phase-separation state with distinct physical properties^45^. We observed that the C-terminal deletion of EBNA2 did not affect the formation of ACDs, but lost the ability to recruit acetyltransferase p300 and was no longer associated with H3K27ac condensates, indicating that the formation of ACDs was independent of histone acetylation. Our results also suggested that EBNA2 has dual functions in epigenetic regulation on the host genome. On the one hand, phase separation of EBNA2 reorganizes chromatin topology to form ACDs. On the other hand, it recruits histone acetyltransferase to promote histone acetylation on accessible chromatin regions and regulate gene expression. The two functions are performed by the N-terminal and C-terminal, respectively.

EBNA2 widely affects the epigenetic characteristics of the host genome. Here we proposed a phase-separation mode to explain the mechanism of EBNA2 in epigenetic regulation. It helps to understand how the Epstein-Barr virus affects human health and develop therapeutics for EBV-related diseases.

## Methods

### Plasmids and constructs

The EBNA2 (strain HONE1) CDS was cloned into a PCW57.1 vector using Gibson assembly. EBNA2 (strain B95-8) was synthesized by Sangon, China. The PCR primer pairs are as follow: 5′-cgatgacaagagcggaggctccgccatgcctacattctatcttgc-3′, 5′-aaaaggcgcaaccccaaccccgttactggatggaggggcgaggtc-3′. The EBNA2 and mutant CDS were cloned into pCAG-scFv-GCN4_V4-mNeonGreen vector (gift from Zhili Rong) and pCAG-scFv-GCN4_V4-mCherry vector by Gibson assembly. The PCR primer pairs are as follow: EBNA2-LCDF: 5′-gtacaagggtggaggtcggaCCatgcctacattctatcttgc-3′ and EBNA2-LCDR: 5′-agcgagctctagcccgggcgtcgactcactggatggaggggcgaggtc-3′. EBNA2(1-176) F: 5′-gtacaagggtggaggtcggaCCatgcctacattctatcttgc-3′ and EBNA2(1-176) R 5′-agcgagctctagcccgggcgtcgactcaTGTTGCAGGTGGCAGAGGGG-3′. EBNA2(△1-176) F: 5′-gtacaagggtggaggtcggaCCatgcctCTAACGGTGCCACCAAGGCC-3′ and EBNA2(△1-176) R: 5′-agcgagctctagcccgggcgtcgactcactggatggaggggcgaggtc-3′. EBNA2(△CTD) F: 5′-gtacaagggtggaggtcggaCCatgcctacattctatcttgc-3′ and EBNA2(△CTD) R: 5′-agcgagctctagcccgggcgtcgactcaGGACTCCGGTTCATGTATTG-3′. EBNA2(△polyRG + △CTD) F: 5′-acaagggtggaggtcggaccatgaagggcaagtcgaGGGA-3′ and EBNA2(△polyRG + △CTD) R: 5′-agcgagctctagcccgggcgtcgactcactggatggaggggcgaggtc-3′. EBNA2(△polyRG) F: 5′- ggccagagccGGGGACAGAGCAAGGGCAAGTCCAGGGACAA-3′ and EBNA2(△polyRG) R: 5′-ttgtccctggacttGCCCTTGCTCTGTCCCCGGCTCTGGCC-3′. EBNA2(△polyRG + CTD) F: 5′- tgtacaagggtggaggtcggaCCatgcctacattctatcttgc-3′ and EBNA2(△polyRG + CTD) R: 5′-ctagcccgggcgtcgactcaGCTCTGTCCCCGGCTCTGGCC-3′. EBNA2(W424T) F1: 5′- AGACTTAGACGAAAGTACCG-3′, EBNA2(W424T) F2: 5′- AGACTTAGACGAAAGTACCGATTACATTTTTGAGAcaaca-3′ and EBNA2(W424T) R: 5′- gcgagctctagcccgggcgtcgactcactggatggaggggcgaggtc-3′. The 3×Flag EBNA2 and mutant CDS were cloned into pCAG-scFv-GCN4_V4-mNeonGreen vector by Gibson assembly. The PCR primer pairs are as follow: 3×Flag F1: 5′-tggcaaagaatttgctagcatggactacaaagaccatgacggt-3′ and 3×Flag R1: 5′- tcatcacgatgtcggggcccatcttgtcatcgtcatccttgtaat-3′. To construct scFv-mNeonGreen-p300 IDR-NLS, p300 IDR was generated by PCR and cloned into the pCAG-scFv-GCN4_V4-mNeonGreen vector. The primer pairs are as follow: p300 IDR F: 5′-gtacaagggtggaggtcggaCCactcctgccactccaacgac-3′ and p300 IDR R: 5′-gataagcttgtactcttcaccgtgtatgtctagtgtactct-3′. To construct scFv-mNeonGreen-CTCF (573-728)-IDR, CTCF-IDR was first PCR amplified using the primers 5′-gtacaagggtggaggtcggaCCcatgctgataattgtgctgg-3′ and 5′-gataagcttgtactcttcaccccggtccatcatgctgaggat-3′ (Fig. S2). To construct scFv-mNeonGreen-EBNA2(1-176)-NLS, SV40 NLS was first PCR amplified using the primers 5′-CCCCTCTGCCACCTGCAACAggtggtggtagcggtggtggtactagtcccaagAAGAAG-3′ and 5′-agcgagctctagcccgggcgtcgactcacaccttgcgcttcttcttgggactagtacc-3′. The amplified EBNA2(1-176), SV40 NLS fragments and pCAG-scFv-GCN4_V4-mCherry vector were then assembled using Gibson assembly. For insertion of the glutamic acid expansions +8E, glutamine expansions +10Q, histidine expansions +10H and glycine expansions +10G, single stranded oligonucleotides encoding the respective amino acid expansions were inserted into the pCAG-scFv-mNeonGreen-EBNA2(1-176)-NLS using Gibson assembly. For the generation of the amino acid expansions-NLS, the primer pairs are as follow: SV40-NLS F: 5′-gaagaagaagaagaagaagaagaagaagaaggtggtggtagcggtggtgg-3′, SV40 NLS R: 5′-agcgagctctagcccgggcgtcgactcacaccttgcgcttcttcttgggactagtacc-3′. +8E SV40-NLS F: 5′-gaagaagaagaagaagaagaagaaggtggtggtagcggtggtgg-3′, EBNA2(1-176) +8E-NLS F: 5′-CCCCTCTGCCACCTGCAACAgaagaagaagaagaagaagaagaagaagaa-3′ and SV40 NLS R: 5′-agcgagctctagcccgggcgtcgactcacaccttgcgcttcttcttgggactagtacc-3′. +10Q SV40-NLS F: 5′-CAACAACAACAACAACAACAACAACAACAAggtggtggtagcggtggtgg-3′, EBNA2(1-176) +10Q-NLS F: 5′-CCCCTCTGCCACCTGCAACACAACAACAACAACAACAACAACAACAACAA-3′ and SV40 NLS R: 5′-agcgagctctagcccgggcgtcgactcacaccttgcgcttcttcttgggactagtacc-3′. +10H SV40-NLS F: 5′-CACCACCACCACCACCACCACCACCACCACggtggtggtagcggtggtgg-3′, EBNA2(1-176) +10H-NLS F: 5′-CCCCTCTGCCACCTGCAACACACCACCACCACCACCACCACCACCACCAC-3′ and SV40 NLS R: 5′-agcgagctctagcccgggcgtcgactcacaccttgcgcttcttcttgggactagtacc-3′. +10G SV40-NLS F: 5′-GGAGGAGGAGGAGGAGGAGGAGGAGGAGGAggtggtggtagcggtggtgg-3′, EBNA2(1-176) +10G-NLS F: 5′-GGAGGAGGAGGAGGAGGAGGAGGAGGAGGAggtggtggtagcggtggtgg-3′ and SV40 NLS R: 5′-agcgagctctagcccgggcgtcgactcacaccttgcgcttcttcttgggactagtacc-3′.

### Cell culture, transfection, and generation of the EBNA2 inducible cells

HEK293T cells or CNE2 S18 cells were cultured at 37 °C and 5%CO2 in complete medium: DMEM supplemented with 10% fetal bovine serum (FBS, Excell FSP500), 100U/ml penicillin/streptomycin (BI 03-050-1A) and 2mM l-glutamine (BI 03-022-1B). No International Cell Line Authentication Committee cell lines were used in this study. HEK293T cells were cultured in a glass-bottom cell culture dish (NEST, 801001). Cells were transfected with Lipofectamine 2000 (Thermo Fisher) following the manufacture’s instruction. The cell culture media were changed 1 h before transfection. After 5 min of incubation, the lipofectamine-plasmid mix was added to the cell media. The cell media was changed after 8 h transfection. The EBNA2 inducible cell lines were generated by Lentivirus transfection and were induced by 10 μg/mL doxycycline for 48 h. The expression of proteins was confirmed by Western blot.

### Live cell imaging

After 24–48 h transfection, cells were imaged by confocal microscope (Carl Zeiss, LSM 880 with Airyscan). Cells were maintained at 37 °C and 5% CO_2_ condition during imaging. ZEN black edition version 2.3 was used for acquisition. Standard culture media was replaced by different concentrations (10, 5, and 2%) of 1,6-hexanediol. 1,6-hexanediol was added to the transfected cells during the live imaging. For live-cell imaging of EBNA2/p300 IDR fusions and fissions, HEK293T cells were cultured in standard conditions. For confocal imaging, the cell was imaged 48 h after transfection on a Nikon A1 R confocal microscope at room temperature with a plan-apochromat ×100/1.40 oil objective.

### Fluorescence recovery after photobleaching (FRAP)

FRAP was performed on Nikon with a 488 nm laser. Using 100% laser power to bleach and images were collected every 2 s. When taking images, we used low laser transmission to avoid photobleaching the entire image. To do the FRAP experiment, take three control images before bleaching. Measure relative fluorescence intensity of the spine of interest (Fs), a transfected but unbleached region (control), and a background region (Fb) in time-lapse images. Calculate the photobleaching rate (*r*) by comparing the fluorescence of the control region before (Fc0) and after (Fc) photobleaching: *r* = Fc/Fc0. Normalize the fluorescence intensity of the target spine (*F*) as follows: *F* = (Fs − Fb)/*r*. Calculate the mobile fraction (fm) by the following equations: fm = F∞/F0.

### Immunofluorescence

Cells were plated on a glass-bottom cell culture Petri dish (NEST,801001) and grew for 24 h followed by fixation using 4% paraformaldehyde (Pierce™ 16% Formaldehyde ((w/v)), Methanol-free, ThermoFisher, 28906) in 1 × PBS for 20 min at room temperature. After washing three times in 1 × PBS, cells were blocked with 4% IgG-free Bovine Serum Albumin for 20 min at room temperature. Permeabilization of cells was performed with 0.2% Triton X-100 (Sigma Aldrich, X100) in 1 × PBS for 10 min, followed by adding primary antibody 1:500 in 1 × PBS and incubating at 37 °C for 2 h. Cells were washed with 1 × PBS three times, followed by incubation with a secondary antibody 1:1000 in 1 × PBS for one hour. After washing three times, the nuclei were stained with 10 µg/ml DAPI (Beyotime). Images were acquired by a confocal microscope (Carl Zeiss, LSM 880 with Airyscan or Nikon A1 R) with a 100× objective. At least three different dishes were quantified per treatment type.

### ATAC-see imaging

HEK293T cells were plated onto 15 mm glass-bottom cell culture dish (NEST, 801002) at 80% confluency with fibronectin coating one hour at 37 °C before the experiment. Cells were fixed with 4% paraformaldehyde (Pierce™ 16% Formaldehyde ((w/v)), Methanol-free, ThermoFisher, 28906) in 1 × PBS for 20 min at room temperature. After fixation, the cells were washed three times in 1 × PBS for 5 min and then permeabilized in ATAC lysis buffer (10 mM Tris-Cl, pH7.4, 10 mM NaCl, 3 mM MgCl, and 0.1% Igepal CA-630) for 10 min at room temperature. After permeabilization, the dishes were washed twice with 1 × PBS and in a humid chamber at 37 °C. The transposase mixture solution (25 μl 2 × TD buffer, a final concentration of 80 nM proteinG-Tn5 ((Vazyme, Nanjing, China)) adding dH2O up to 50 μl) was added to the cells. The sample was incubated in a humidity chamber for 30 min at 37 °C. After the transposase reaction, dishes were washed with PBS containing 0.01% SDS and 50 mM EDTA for 15 min three times at 55 °C. After washing, cells were incubated with the secondary antibody labeled with DyLightTM 549 (1:200; rabbit, 610-442-002, ROCKLAND) for 1 h at room temperature. Dishes were washed twice with 1 × PBS and incubated with DAPI (1:1000, C1002, Beyotime) for 15 min at room temperature and then imaged using a Nikon A1 R confocal microscope. At least three different dishes were quantified per treatment type.

### Western blot

Proteins were extracted using the RIPA solution. Proteins were separated on a 10% sodium dodecyl sulfate-polyacrylamide gel electrophoresis (SDS-PAGE) and transferred to a PVDF membrane. The primary antibodies against GAPDH (10494-1-AP, Proteintech, USA), HA-Tag (C29F4, Cell Signaling Technology, USA), Histone H3 (ab1791, Abcam, USA), Histone H3 (acetyl K27) (ab4729, Abcam, USA) and FLAG (66008-3-1 g, proteintech, USA) were diluted according to the instructions.

### RNA extraction and quantitative real-time PCR analysis

Total RNA from Raji/HEK 293T (transfected with EBNA2) was extracted using Trizol reagent (Vazyme, China), according to instructions provided by the manufacturer. Total RNA (1 μg) was then used for reverse transcription (RT) with a commercially available kit (Vazyme, China). Real-time polymerase chain reaction (PCR) was performed in triplicate with an ABI Step One Plus system (Applied Biosystems, USA) and a fluorescence-labeled SYBR Green/ROX qPCR Master Mix kit (Yeasen, China) using specific primers. EBNA2 primers for RT-qPCR are EBNA2 F: 5′-CCCATCCAATGCCGCCCCCG-3′, EBNA2 R: 5′-gaggtcttttactgggtccc-3′, and glyceraldehyde-3-phosphate dehydrogenase (GAPDH) primers for RT-qPCR are homo GAPDH RT-PCR F: GGAGCGAGATCCCTCCAAAAT, homo GAPDH RT-PCR R: GGCTGTTGTCATACTTCTCATGG, which was used as an endogenous control were detected. For quantification, the point of product accumulation in the early logarithmic phase of the amplification plot was defined by assigning a fluorescence threshold above the background, defined as the threshold cycle (Ct) number. Relative expression of different gene transcripts was calculated by the ΔΔCt method. The Ct of any gene of interest was normalized to the Ct of the normalizer (GAPDH). Fold changes (arbitrary units) were determined as 2−ΔΔCt.

### Colocalization

Colocalization of two channels was done using the ImageJ Plot Profile tool.

### Chromatin immunoprecipitation sequencing (ChIP)

ChIP-seq was performed as previously described^50^. Briefly, 1 × 10^6^ CEN2 cells were washed with cold 1 × PBS twice. Washed cells were then lysed in 200 μl of Cell Lysis Buffer (80 mM NaCl, 10 mM Tris-HCl pH8.0, 10 mM sodium butyrate, 6 mM MgCl2, 1 mM CaCl2, 250 mM sucrose, 0.02% NP-40, freshly added 0.1 mM PMSF and 1 × protease inhibitor cocktail) for 5 min on ice. Nuclei were pelleted and washed once with 200 μl of Nuclei Washing Buffer (10 mM NaCl, 10 mM Tris-HCl pH8.0, 10 mM sodium butyrate, 3 mM MgCl_2_, 1 mM CaCl_2_, and 250 mM sucrose). Digestion was then performed by incubation for 5 min at 37 °C in 200 μl of the same buffer containing 6U micrococcal nuclease (Worthington). The reaction was stopped by the addition of 8 μl of solution containing 0.2 M EDTA and 0.2 M EGTA. After centrifuge for 10 min at 18,000 × *g*, the supernatant was dilute into a final concentration of 16.7 mM Tris pH 8.0, 1.2 mM EDTA, 167 mM NaCl, 1.1% TritonX (vol/vol),0.1 mM PMSF, and 1 × protease inhibitor cocktail. Chromatin was then incubated overnight at 4 °C with anti-trimethylated H3K27 antibody (Millipore, 07-449, 1:100 dilution) or control rabbit IgG (R&D Systems, ab-105-c, 1:100 dilution). Protein A/G magnetic beads (Pierce,88802) were added for an additional 4 h of incubation. Immunoprecipitates were washed vigorously and DNA was purified with a QiaQuick PCR purification kit (Qiagen). After end repair, dA-tailing, and linker ligation, barcodes, and Illumina adapters were then added to ChIP products and. The amplified libraries were purified with a QiaQuick PCR purification kit and size selection by 0.7× and 0.2× Ampure XP beads (Beckman, A63880). ChIP-seq libraries were sequenced via paired-end 150 bp reads on a HiSeq X TEN platform (Novogene, China).

### Cleavage under targets and tagmentation (CUT&Tag)

CUT&Tag was carried out following the manufacture’s instruction (Vazyme Biotech, TD901-01). In brief, transient transfection of EBNA2 and vehicle plasmid into BJAB cells respectively, collect positive cells by fluorescence-activated cell sorting (FACS) after 48 h. Approximately 100,000 BJAB cells were processed by centrifugation between buffer exchanges at 600 × *g* for 3 min and in a swinging bucket rotor at room temperature for the initial wash and incubation steps. Cells were collected and washed with 1 ml of Wash Buffer at room temperature. Cells were rotated and incubated in antibody (ab4729, Abcam, USA) diluted 1:50 in Antibody Buffer for 2 h at room temperature. Permeabilized nuclei were then centrifuged once and incubated with anti-rabbit IgG antibody (FD0218, Fudebio, China, 1:100 dilution) in 1 ml of Dig-wash Buffer on a rotator at room temperature for 1 h. Nuclei were washed twice with Dig-wash Buffer and incubated with 1:100 dilution of pG-Tn5 in Dig-300 Buffer for 1 h at room temperature on a rotator. Cells were washed two times with Dig-300 Buffer and resuspended in 300 µl of Tagmentation Buffer and incubated at 37 °C for 1 h. Tagmentation was stopped by adding Stop Buffer (each sample with 10 μl 0.5 M EDTA, 3 μl 10% SDS, and 2.5 μl 20 mg/ml Proteinase K), and the sample was held for 1 h at 50 °C in an incubator. The remaining steps are carried out according to the product manual.

### Omni-ATAC-seq

Transient transfection of EBNA2 and vehicle plasmid into BJAB cells respectively, collect positive cells by fluorescence-activated cell sorting (FACS) after 48 h. To profile open chromatin, a total of 5 × 10^4^ cell pellets were washed once with cold PBS, cells lysed on ice for 3 min in 50 μL ice-cold Lysis Buffer (10 mM Tris pH 7.4, 10 mM NaCl, 3 mM MgCl_2_, 0.1% NP-40, 0.1% TWEEN 20, and 0.01% Digitonin in DEPC H_2_O), resuspended in 1 mL ice-cold RBS-Wash (10 mM Tris pH 7.4, 10 mM NaCl, 3 mM MgCl_2_, and 0.1% TWEEN 20) and pelleted at 4 °C at 500 × *g* for 10 min. Tagmentation was performed in 1 × Tagmentation Buffer (10 mM Tris pH 7.4, 5 mM MgCl_2_, 10% DMF, 33% PBS, 0.1% TWEEN 20, and 0.01% Digitonin) using 100 nM Tn5 Transposase for 30 min at 37 °C. Tagmentation was inactivated with the addition of five volumes of SDS Lysis Buffer (100 mM Tris pH 7.4, 50 mM NaCl, 10 mM EDTA, and 0.5% SDS in H_2_O) and 100 μg Proteinase K (Invitrogen, 25530015, USA) for 30 min at 55 °C. Centrifuge at 500 × *g* to take the supernatant, DNA was size selected and purified using QIAquick PCR Purification Kit (QIAGEN, 28106, GERMANY) according to the manufacturer’s instructions.

### Hi-C procedure

HEK 293T cells were collected after 48 h transfection. Digestion with the MboI enzyme, filling-in with biotin-labeled dCTP, and re-ligation by the T4 ligase were performed using fixed cells (10 × 10^6^ cells) following the instructions of the in situ Hi-C method. After ligation, cells were degraded by protein K. DNA was precipitated by isopropanol, then dissolved by 130 μL of 1 × Tris buffer (10 mM Tris-HCl, pH 8), Sonicated on 60% power for 15 s on/25 s off for 15 min at 4 °C (Q800R2 sonicator). Took out 5 μg of sonicated DNA. 5 μL of 10 mg/ml Dyna beads MyOne Streptavidin C1 beads (Invitrogen, 65001) was washed with 200 μL of 1 × Tween Washing Buffer (1 × TWB: 5 mM Tris-HCl (pH 7.5); 0.5 mM EDTA; 1 M NaCl; 0.05% Tween 20). The beads were separated on a magnet, the solution was discarded. The beads were resuspended in 100 μL of 2 × Binding Buffer (2 × BB: 10 mM Tris-HCl (pH 7.5); 1 mM EDTA; 2 M NaCl), added to sheared DNA, incubated at room temperature for 15 min with rotation to bind biotinylated DNA. The ends of sheared DNA were repaired and the biotin from un-ligated ends was removed, adapters were added to the A-tailed DNA fragments following in situ Hi-C protocol. PCR was performed with thirteen to nineteen cycles using Illumina primers. Finally, DNA size selection was performed with 0.55–0.75x volume of VAHTS DNA Clean beads (Vazyme, N411-01-AA) to make sure the DNA length distributes between 300 and 500 bp. The library was quantified with Qubit and sequenced using Novaseq-PE150 Illumina sequencing platform at Berry Genomics Corporation Inc.

### Analysis of ATAC-Seq data

Raw sequence reads were initially processed for removing adapter sequences and poor quality reads by Fastp (v 0.20.0). Subsequently, the remaining reads were mapped to the human genome hg38 using Bowtie2(v2.3.5.1) with parameters (-sensitiv, -X 2000). PCR duplicated fragments were filtered by Picard(v2.22.8). Then, we filtered the unmapped, multi-mapped reads and mapping to the reads on chrM. FRiP (fragments ratio in peaks) value was calculated by using bedtools (v2.29.2) and awk (v4.0.2). We used deepTools to generate bigWig file with RPKM normalization, and these files can be visualized in IGV. SAM files were converted to BAM format using Samtools and used for peak calling. MACS2(v2.2.4) with parameters (-t input_file –q 0.01 -f BAM -nomodel -shift −100 -extsize 200 -keep-dup all) was used to call peaks.

### Analysis of CUT&Tag data

Fastp(v 0.20.0) with parameter ‘-thread 8 -5 -3 –W 4’ was used to remove the adapter and low-quality reads. Align paired-end reads used Bowtie2(v2.3.5.1) with the following parameters:-p 8, -sensitive. Duplicated reads were removed using Picard(v2.22.8) with this parameter: REMOVE_DUPLICATES = true. Then, we filtered the unmapped, multi-mapped reads and mapping to the reads on chrM. FRiP (fragments ratio in peaks) value was calculated by using bedtools(v2.29.2) and awk(v4.0.2). We used deepTools to generate bigWig file with RPKM normalization, and these files can be visualized in IGV. Peak calling used HOMER that contains a program called findPeaks with parameters (–style histone). Enriched peaks region generated by HOMER software was used as input to DESeq2(v1.30.0) to find differential peaks from CUT-Tag data as well as normalized the data.

### Analysis of Hi-C data

All Hi-C libraries were sequenced either on an Illumina Hiseq2000 (150 bp paired-end reads). For each sample, reads were obtained following quality filtering and adaptor trimming using fastp (version 0.20.0) with parameter ‘-thread 8 -5 -3 –W 4’. Hi-C mapping, filtering, correction, and binning were performed with the HiC-Pro(v2.11.1) software (https://github.com/nservant/HiC-Pro). The paired-end reads were mapped to the UCSC human genome assembly(hg38). Singleton, multi-mapped, low-quality, unmapped, dumped, dangling, self-circle paired-end reads, and PCR duplicates were all removed by HiC-Pro after mapping. We generated raw contact matrices at 10 kb, 20 kb, 50 kb, 100 kb, 500 kb, 1 Mb resolutions. For raw contact matrices correction from Hi-C data, we used the iterative correction method(ICE) through HiC-Pro software. The hicpro2juicebox.sh utility was used to convert the allValidPairs output of the pipeline into Juicebox.hic format at fragment resolution. Visualization of Hi-C contact matrices was done via juicerbox (https://github.com/aidenlab/juicer/wiki/Download).

### Analysis of ChIP-Seq data

High-confidence reads of ChIP-Seq data obtained by using fastp with default parameters, were mapped to mouse genome mm10 by using Bowtie2 with parameters (-sensitive, –p 6), and PCR duplicated fragments were filtered by Picard. Then, we filtered the unmapped, multi-mapped reads and mapping to the reads on chrM. FRiP (fragments ratio in peaks) value was calculated by using bedtools (v2.29.2) and awk (v4.0.2). We used deepTools to generate bigWig file with RPKM normalization, and these files can be visualized in IGV. Peaks were identified by HOMER that contains a program called findPeaks with parameters (–style histone). Enriched peaks region generated by Homer software was used as input to DESeq2(v1.30.0) to find differential peaks from ChIP-Seq data as well as normalized the data.

### Annotation of ChIP peak sets

To obtain a peak set per condition, we first overlapped the peaks in each replicate and then only the peaks present in both replicates were considered. We used the ChIPSeeker library to annotate the peak sets obtained. Annotation packages: “TxDb.Hsapiens.UCSC.hg38.knownGene” and “org.Hs.eg.db” (Bioconductor). Promoters were defined as ±3 kb from the transcription start site. Venn diagrams were generated using Intervene. Heatmaps and average profiles were performed on bigWig files using deepTools plotHeatmap.

### Identification of chromatin loops

Chromatin loops in CNE2 S18 cells control and CNE2 S18 cells EBNA2+ cells were called using Fit-Hi-C(v2.0.7). First, input files of Fit-Hi-C were created by using a publicly available script(hicpro2fithic.py) from HiC-Pro. Next, for Fit-Hi-C, loops were called using fixed-size bin resolutions from 10 to 25 kb in both cell types. Briefly, significant interaction loops (*q* *≤* 0.05) were identified through jointly modeling the contact probability using raw contact frequencies and ICE normalization vectors with the Fit-Hi-C algorithm.

### Meta-analysis of loops

The popular format to store Hi-C data,.hic, can be converted into.cool files using hic2cool(v0.8.3) software (https://github.com/4dn-dcic/hic2cool). Hi-C matrices in cool format were used to generate genome-wide aggregate plots at loops of HEK293T cells control and HEK293T EBNA2+ cells detected by Hi-C. We used coolpup.py(v0.9.5) to pile-up normalized Hi-C signals at a 25 kb resolution at loops previously identified, and plotted 600 kb upstream and downstream of the loop anchor coordinates(https://github.com/open2c/coolpuppy). We plotted them using plotpup.py(v0.9.5).

### Motif analysis

To analyze the enriched motifs in the peaks from CUT&Tag data, we used the findMotifsGenome.pl to identify enriched motifs from HOMER software.

### Statistics and reproducibility

At least three independent experiments were carried out unless otherwise stated. Statistical analyses were done using the GraphPad Prism software. Statistical significance calculations comparing two conditions were performed using a two-tailed unpaired Student’s t-test.

### Reporting summary

Further information on research design is available in the Nature Research Reporting Summary linked to this article.

## Supplementary information

Supplementary Information

Description of Additional Supplementary Files

Supplementary Movie 1

Supplementary Movie 2

Supplementary Movie 3

Supplementary Data 1

Reporting summary

## Data Availability

The authors confirm that all relevant data are included in the paper and/or its supplementary information files. Original scans for Western blots are provided in Supplementary Fig. 13 and source data files are provided in Supplementary Data 1. The ATAC-seq, ChIP-seq, and Hi-C data have been uploaded to the Gene Expression Omnibus (GEO) with accession code: GSE158288.
