# Supplementary material for: Phase separation of Epstein-Barr virus EBNA2 protein reorganizes chromatin topology for epigenetic regulation
Source: Commun Biol. 2021 Aug 16;4:967. doi: 10.1038/s42003-021-02501-7 (PMC8368186; doi:10.1038/s42003-021-02501-7)
Supplement: Supplementary file 1 — Supplementary Information [file 42003_2021_2501_MOESM1_ESM.docx]

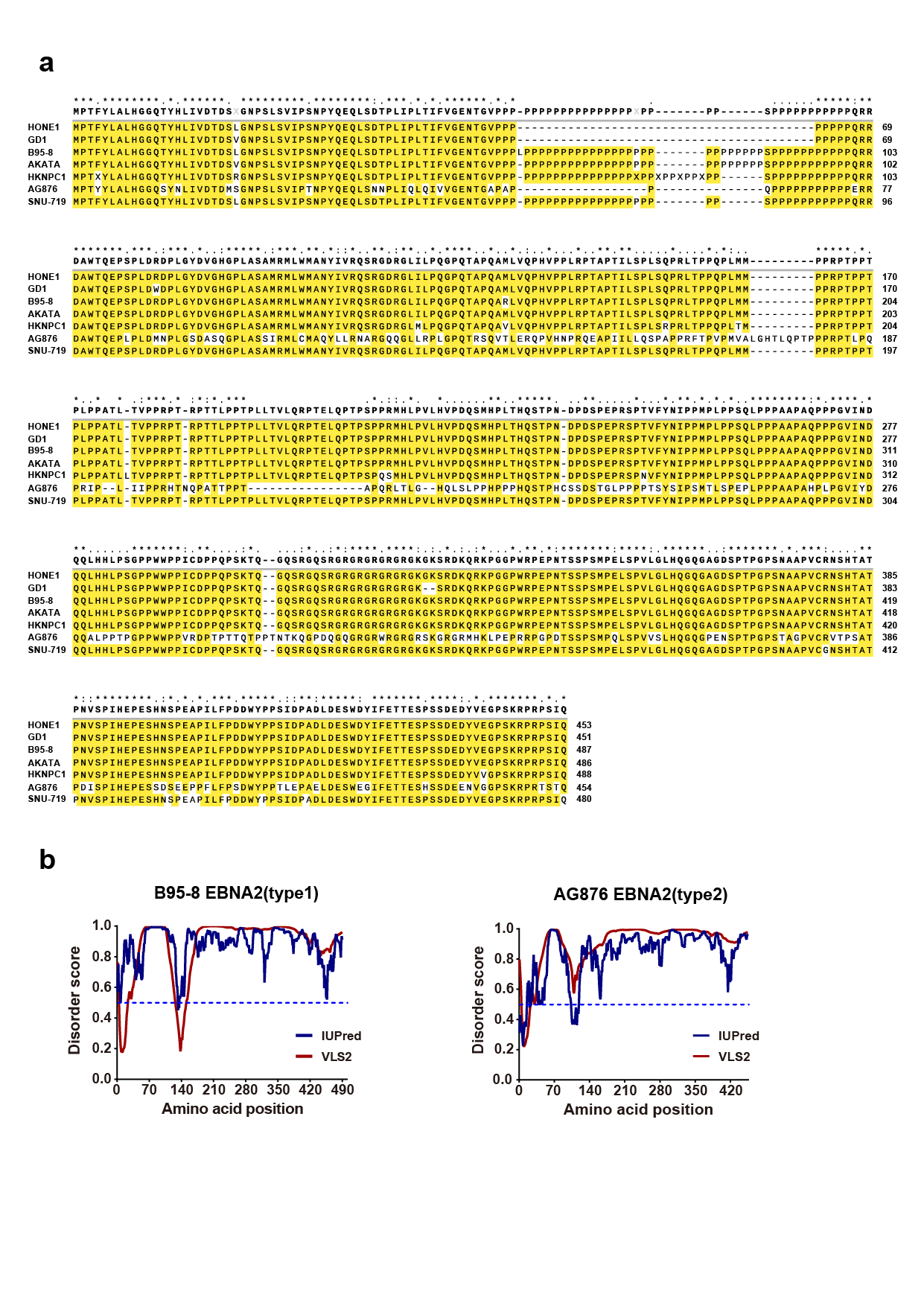


**Fig S1. Multiple sequence alignments of EBNA2 protein sequences of six EBV trains.**

**a.** Yellow positions represent conserved residues among the compared sequences. We used the EBNA2 protein of the laboratory EBV strain HONE1 (type1), which encodes 453 amino acids. The alignment was calculated on the COBALT server. (<https://www.ncbi.nlm.nih.gov/tools/cobalt/>)

**b.** Disorder sequence analysis of B95-8 (type1) and AG876 (type2) EBNA2 protein using the algorithms IUPred (blue) and VSL2 (magenta). IUPred and VSL2 scores are shown on the *y* axis, and amino acid positions are shown on the *x* axis. Dotted line indicates 0.5 disordered score.


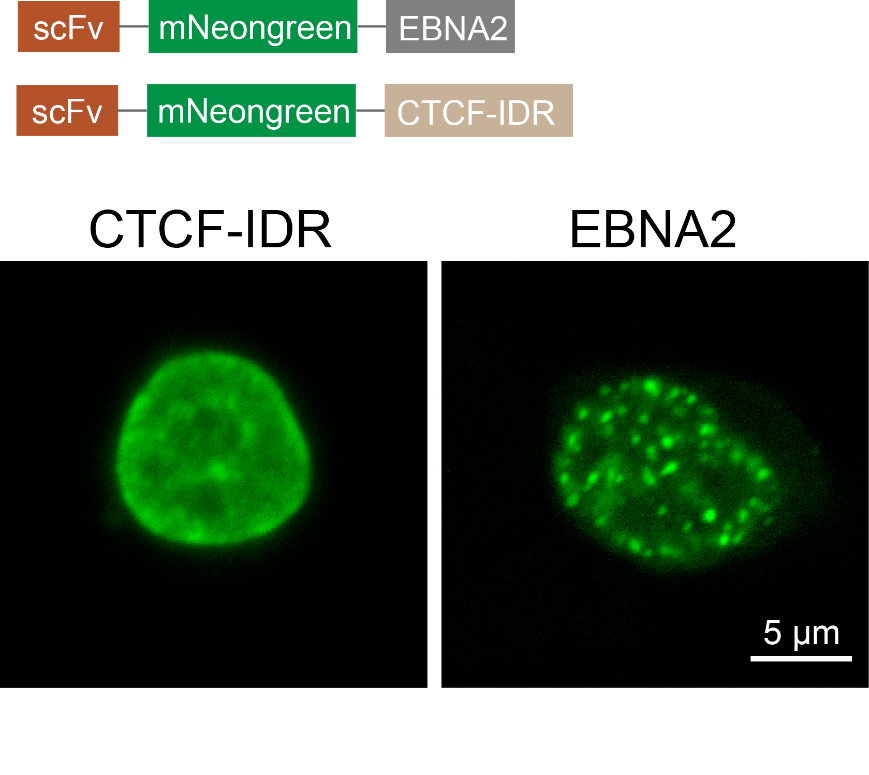


**Fig S2. EBNA2 forms a liquid-like puncta in the nucleus.** Schematic representation for the scFv-mNeonGreen-CTCF-IDR and scFv- mNeonGreen-EBNA2 plasmid (upper). Immunofluorescence images of mNeonGreen-CTCF-IDR (acid amino from 573 to 728) and scFv- mNeonGreen-EBNA2 plasmid (lower). Scale bar, 5μm.

**
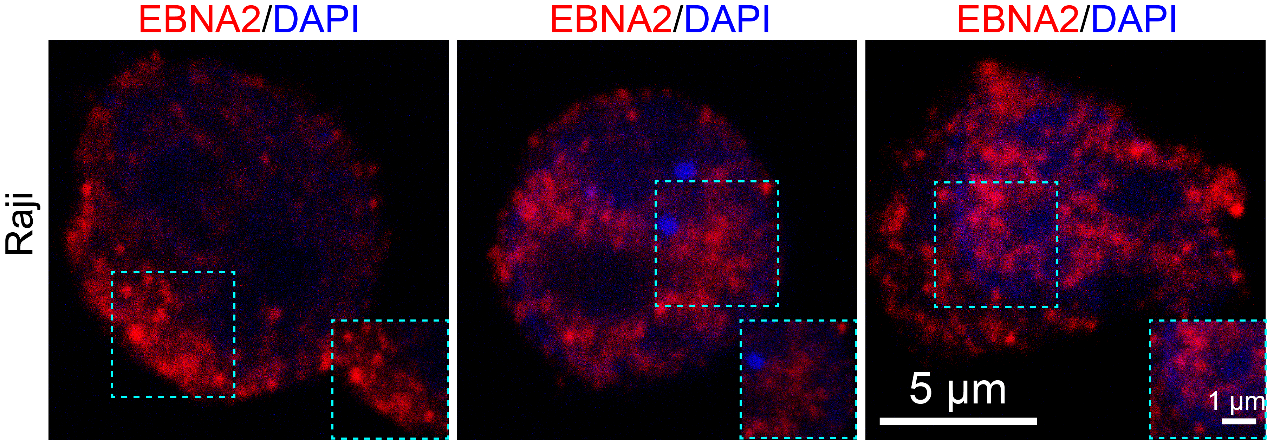
**

**Fig. S3 Immunofluorescence imaging of EBNA2 in EBV-latent infected human Burkitt lymphoma Raji cells.** The fluorescence signal is shown merged with DAPI. Scale bar, 5μm. Magnified region scale bar, 1μm.


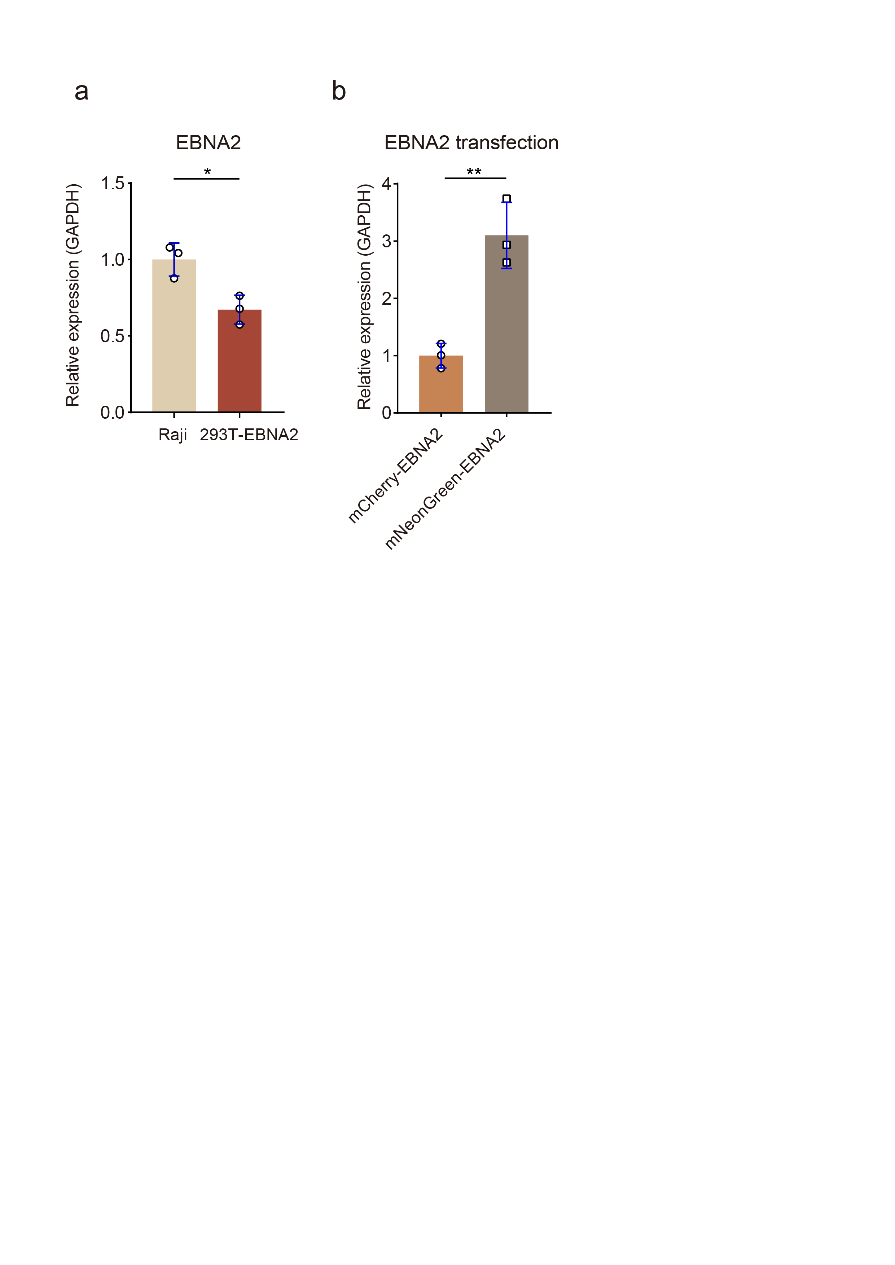


**Fig S4. Detection of EBNA2 expression by q-PCR. a.** Detection of EBNA2 protein expression in Raji cells and 293T-EBNA2 stable cells by q-PCR. **b**. EBNA2 mRNA expression in mCherry-EBNA2 and mNeonGreen-EBNA2 transfected HEK293T cells. Error bars represent SD.


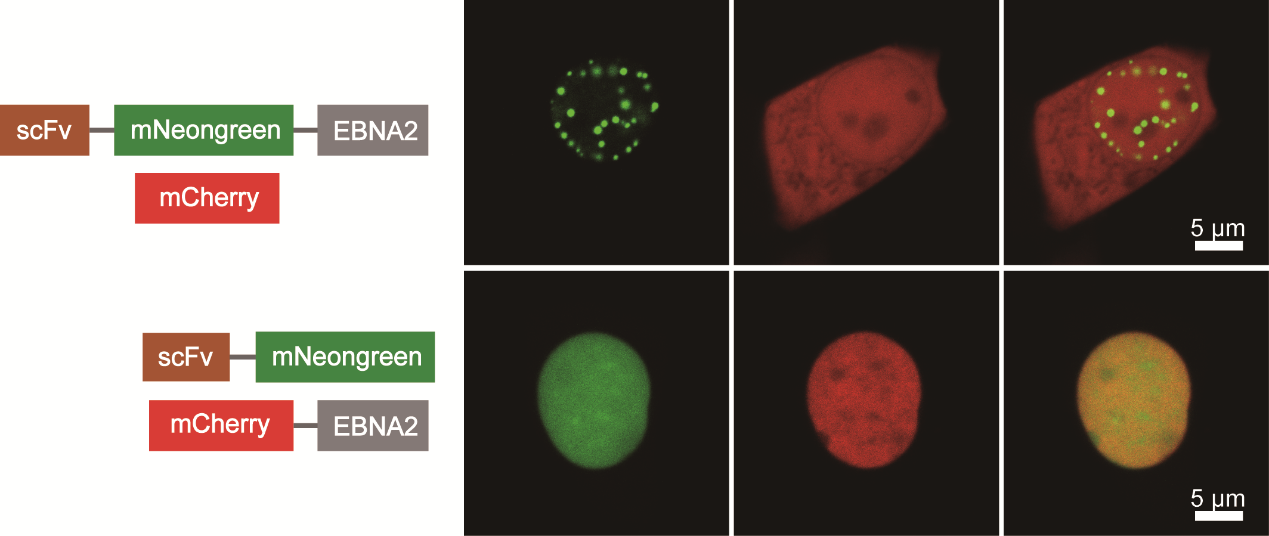


**Fig. S5. EBNA2 self-association.** Schematic representation of recombinant mNeonGreen/mCherry fusion proteins used in this experiment (left). The mNeonGreen-EBNA2 and mCherry (upper) or mNeonGreen and mCherry-EBNA2 (lower) were transiently co-transfected into HEK 293T cells. Double immunofluorescence revealed that mCherry-EBNA2 could not form foci when co-transfection with mNeonGreen proteins in HEK 293T cells (right). Scale bar, 5μm.


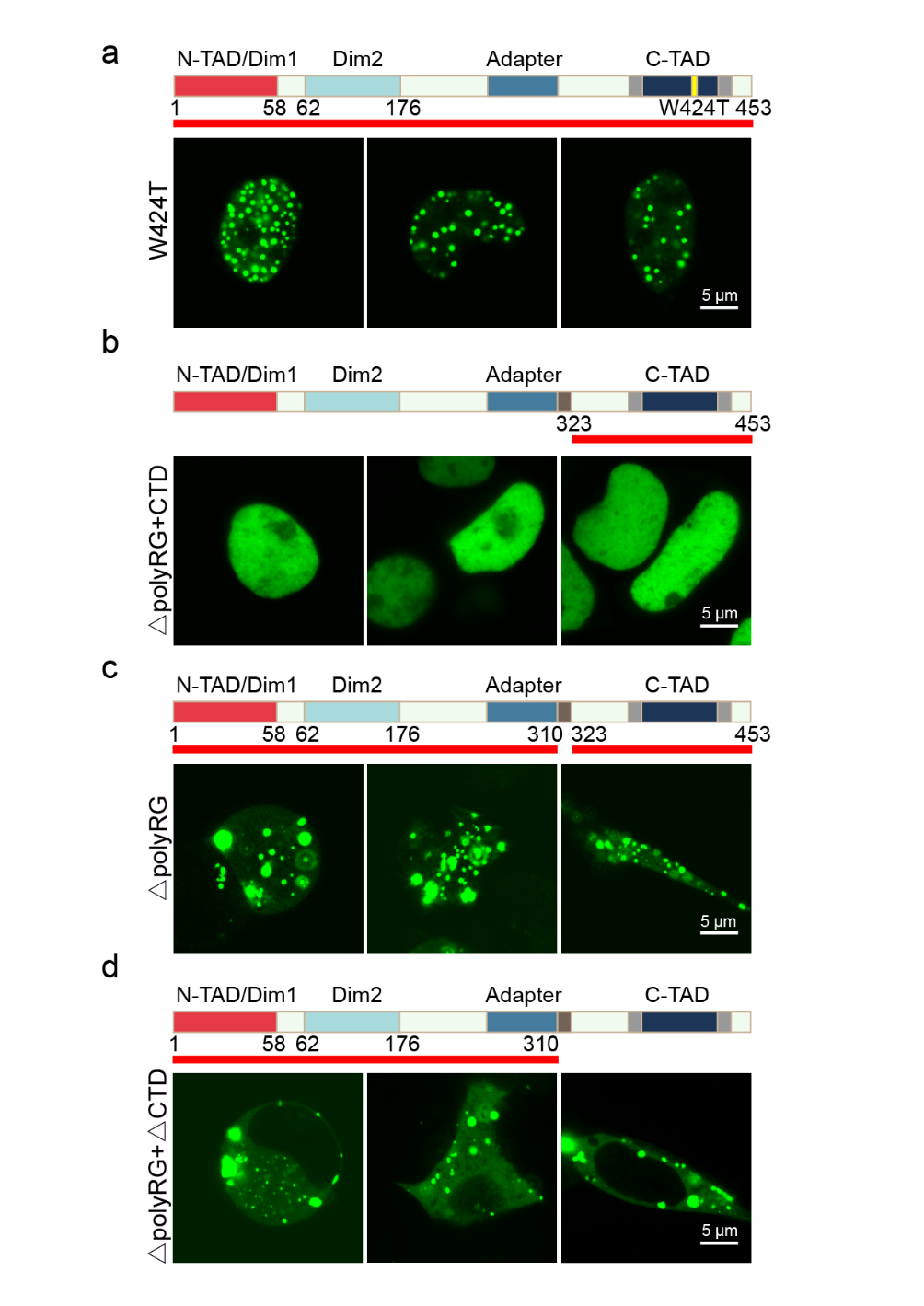


**Fig S6. The N-terminal of EBNA2 is necessary for phase separation. a-d.** Diagram of the truncated EBNA2 (above) and immunofluorescence images of the truncated EBNA2 in HEK293T cells (below). Scar bar, 5μm.


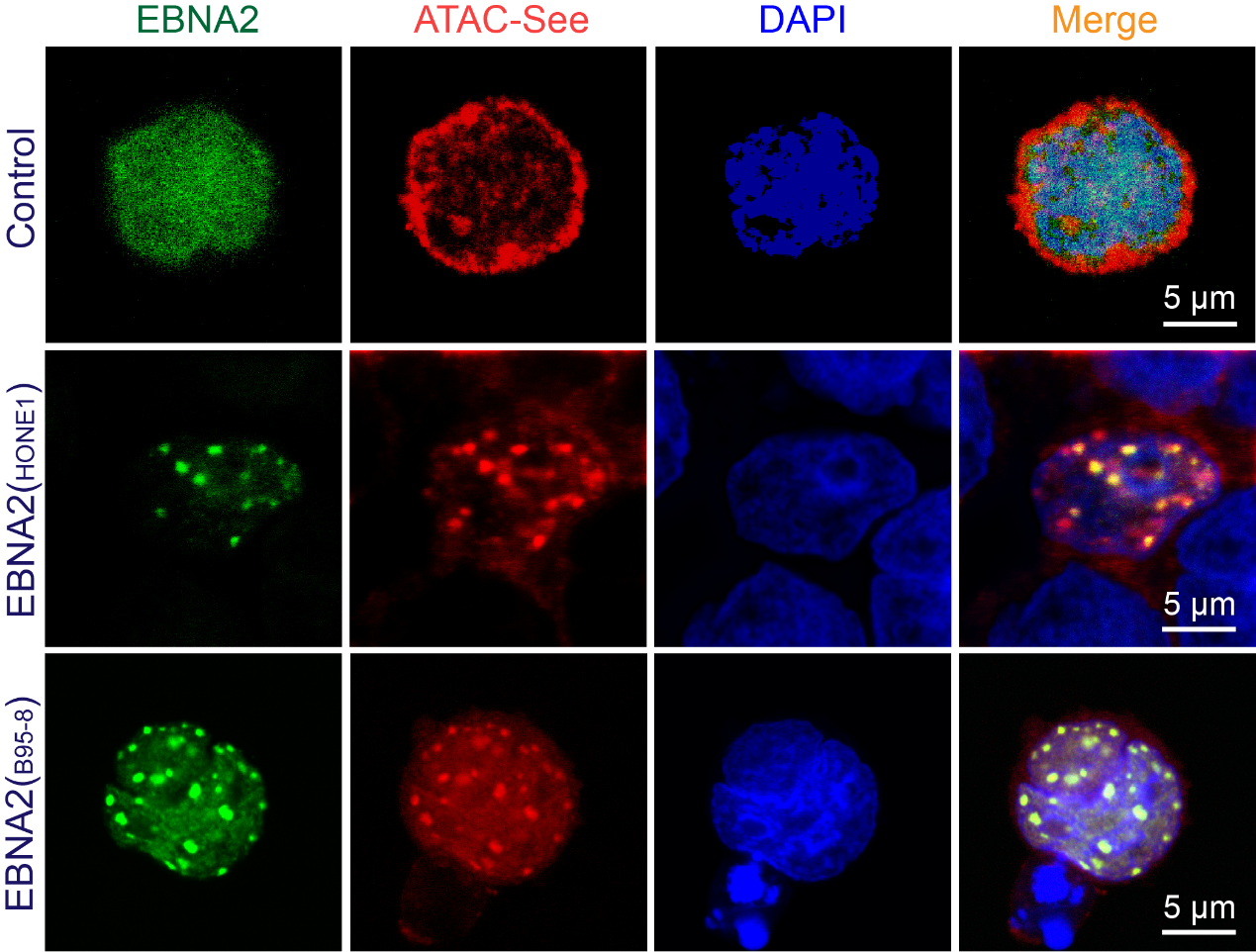


**Fig S7. EBNA2 reorganized chromatin topology to form accessible chromatin domains in BJAB cells.** Representative images of ATAC-see results in BJAB cells. Immunofluorescence images show the co-localization of the accessible chromatin clusters and EBNA2 condensates in BJAB cells. Scar bar, 5μm.

**
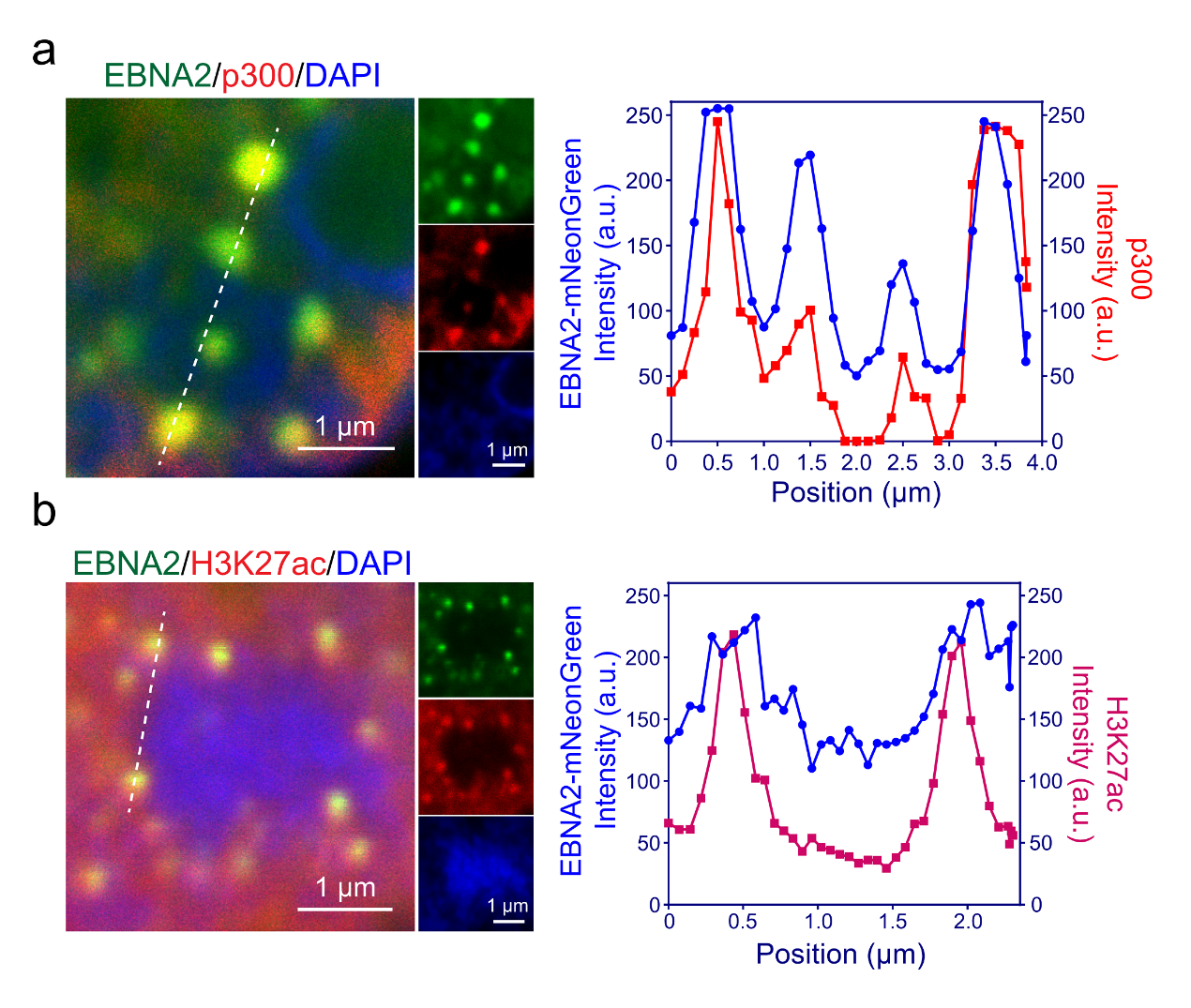
**

**Fig. S8. EBNA2 condensates were associated with p300 and H3K27ac foci. a**, Representative immunofluorescence images showing colocalization of nuclei mNeonGreen-EBNA2 condensates with endogenous p300 in 293T cells. Immunofluorescence images (left) in Fig. 6f magnification of the box region and line plot of the dotted line in the magnified image (right). **b**, Immunofluorescence imaging showing colocalization of mNeonGreen-EBNA2 condensates with H3K27ac in HEK 293T cells. Immunofluorescence images (left) in Fig. 6g magnification of the box region and line plot of the dotted line in the magnified image (right). Scale bar, 1μm.


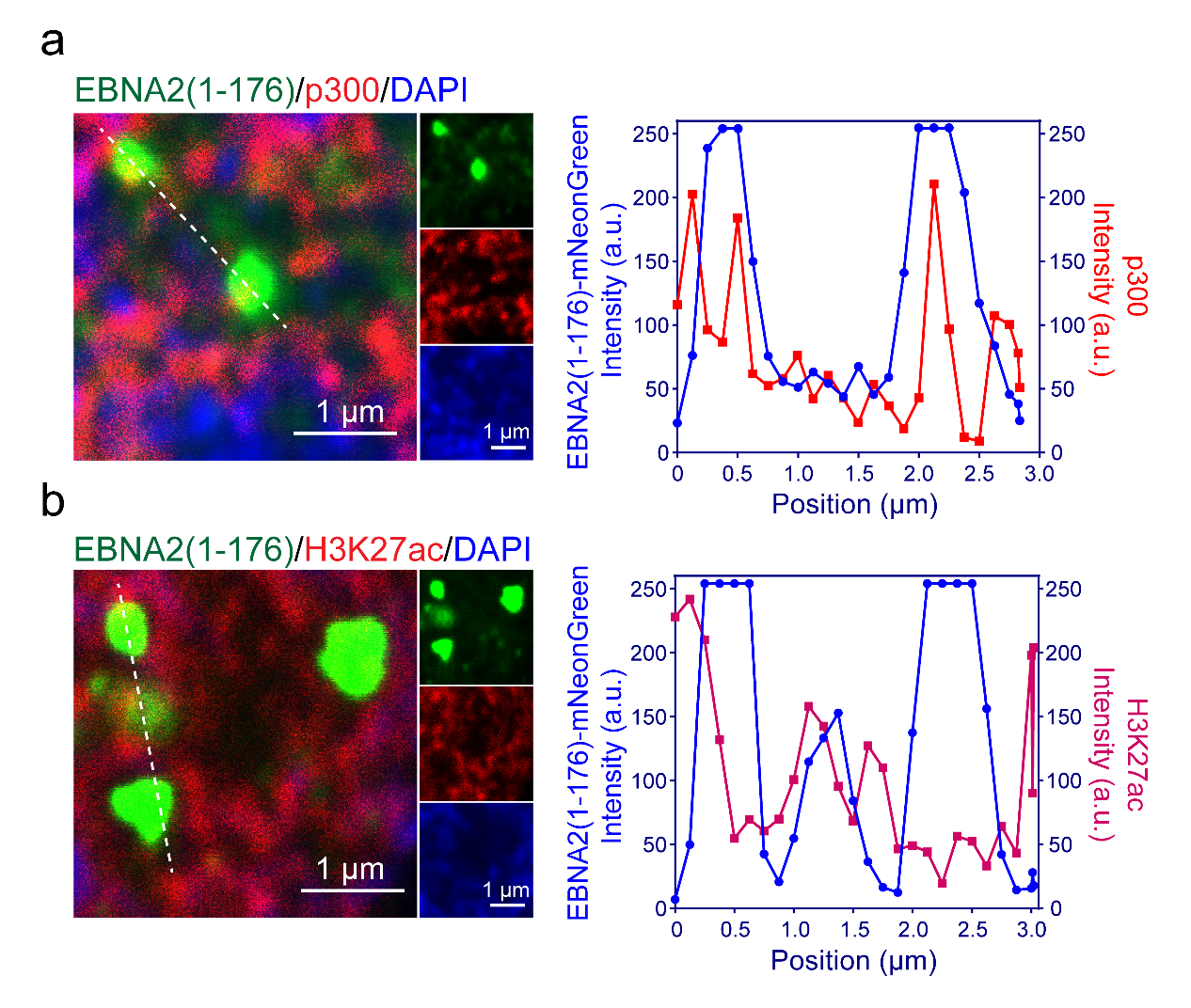


**Fig. S9. EBNA2(1-176) condensates were associated with p300 and H3K27ac foci. a**, Immunofluorescence imaging showing colocalization of mNeonGreen-EBNA2(1-176) condensates with endogenousp300 in HEK 293T cells. Immunofluorescence images (left) in Fig. 6h magnification of the box region and line plot of the dotted line in the magnified image (right). **b**, Immunofluorescence imaging showing no colocalization of mNeonGreen-EBNA2(1-176) condensates with H3K27ac foci in HEK 293T cells. Immunofluorescence images (left) in Fig. 6h magnification of the box region and line plot of the dotted line in the magnified image (right). Scale bar, 1μm.

**
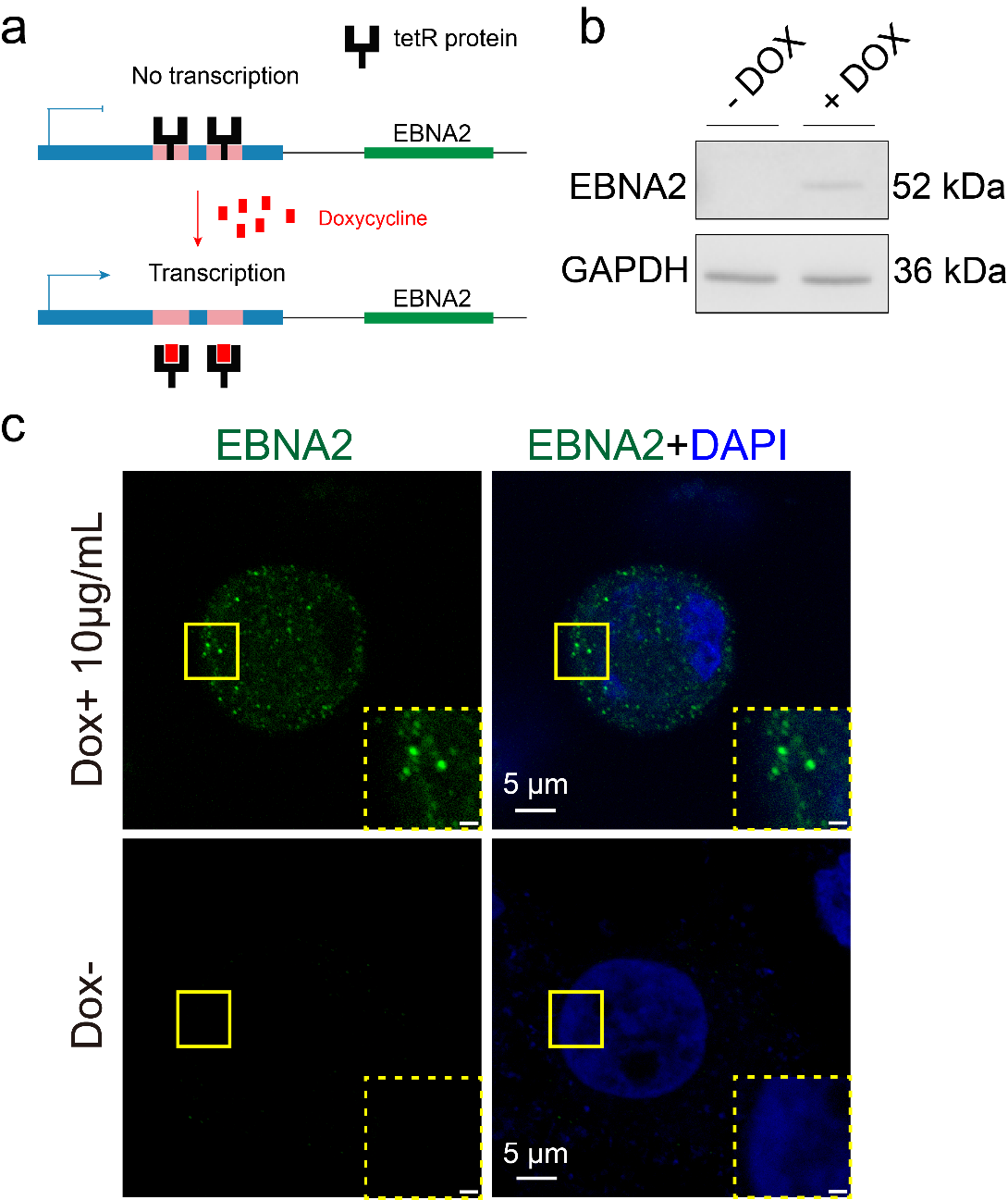
**

**Fig. S10. Western blot and Immunofluorescence assay of EBNA2 Tet-On CNE2 cells. a**, Schematic representation for EBNA2 Tet-On expression system. **b**, Western blot of EBNA2 and GAPDH (loading control) in EBNA2 Tet-On CNE2 cells after 24 hours induction with doxycycline (+Dox) or without doxycycline (-Dox). **c**, Immunofluorescence images of EBNA2 Tet-On CNE2 cells after 24 hours incubation of doxycycline (10μg/mL). Scale bar, 5μm. Magnified region scale bar, 1μm.


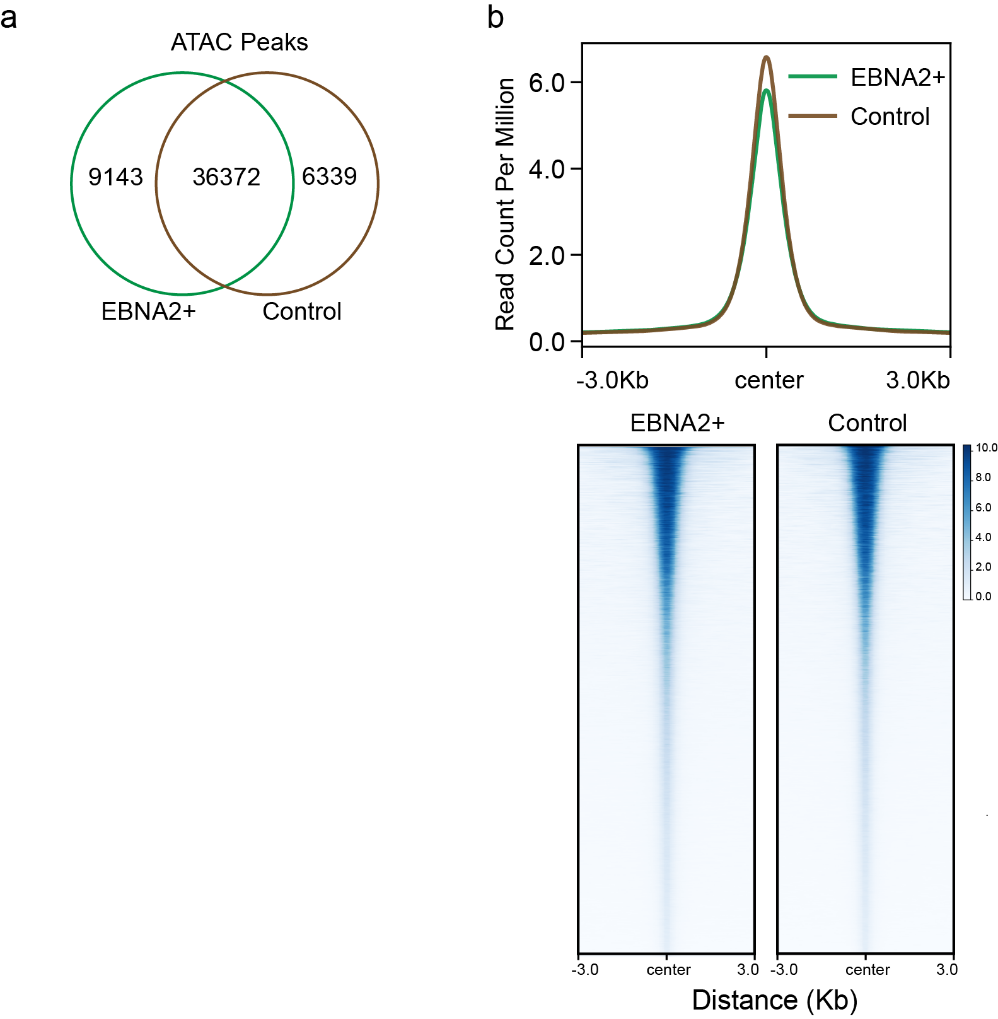


**Fig. S11. Chromatin accessibility in EBNA2+ BJAB cells and control cells. a,** Venn diagrams of open chromatin peaks in EBNA2+ BJAB cells (green track) and control cells (brown track). **b**, The plot and heatmap of open chromatin peaks in the EBNA2+ BJAB cells and control cells.


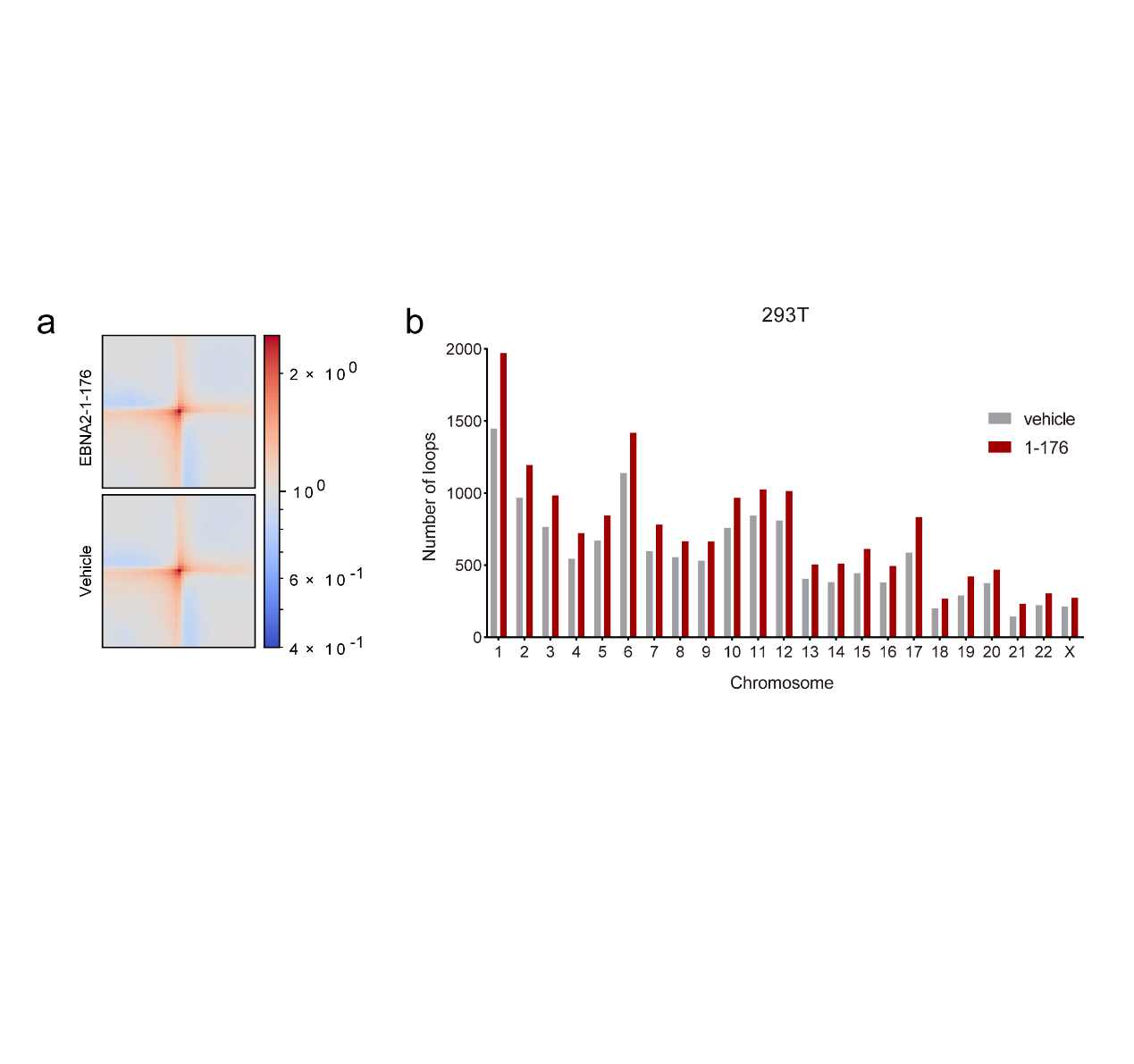


**Fig S12. EBNA2 promotes interactions in genome-wide. a.** The heatmap of aggregated peaks in Hi-C experiments of HEK 293T cells expressing EBNA2 1-176 truncate (upper) and controls (lower). **b.** The distribution of the chromatin loops from Hi-C experiments in each chromosome.


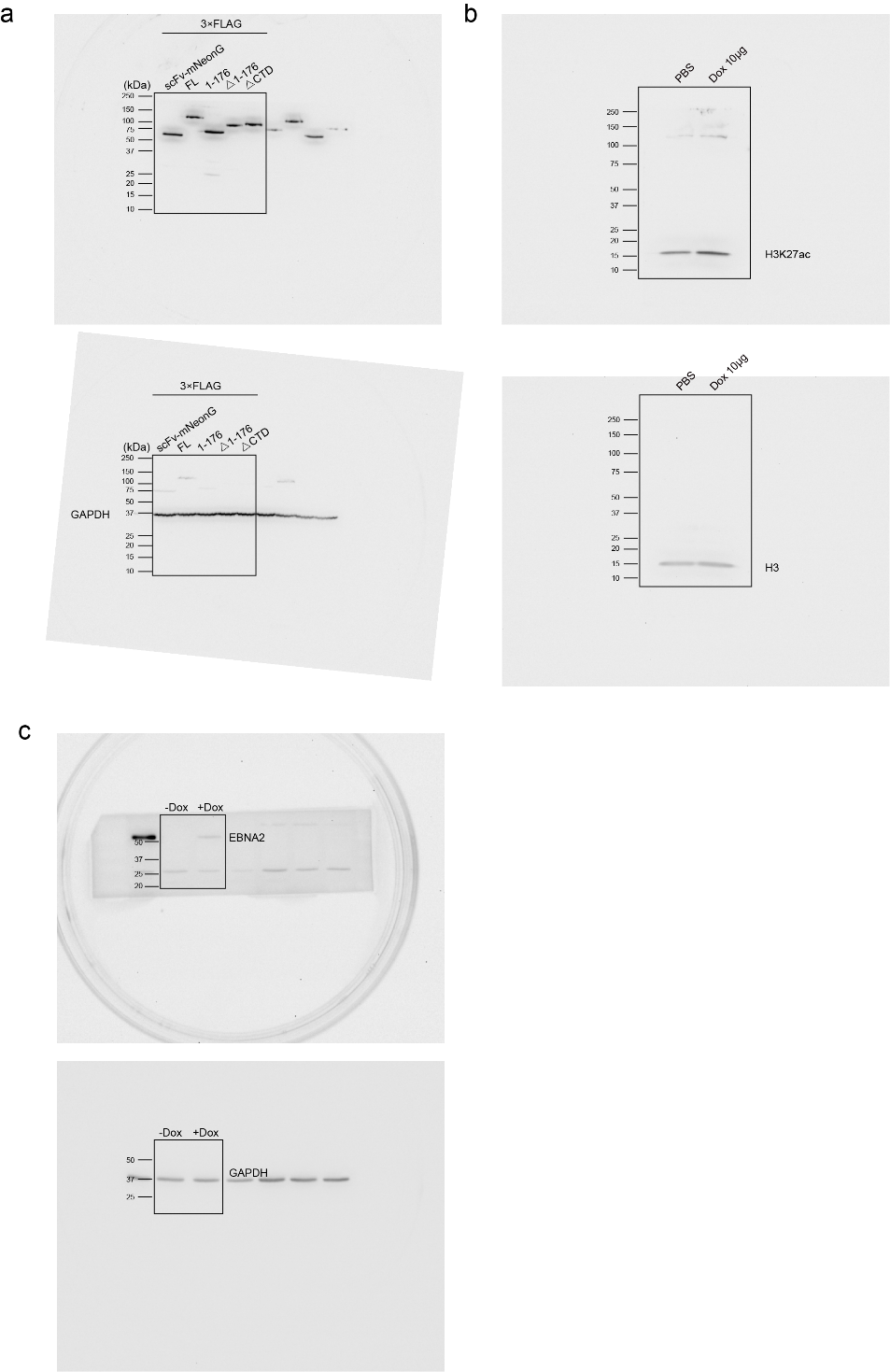


**Fig S13. Non-cropped Western blot images. a.** Non-cropped anti-Flag immunoblotting for Flag-tagged full length and truncates of scFv-mNeonGreen EBNA2 protein (above) and GAPDH (below) in **Figure 3b**. **b.** Non-cropped western blot image of H3K27ac (above) and H3 (below) in **Figure 7f**. **c.** Non-cropped western blot image of EBNA2 (above) and GAPDH (below) in **Supp.** **Figure 10b**.

Movie 1. scFv-mNeonGreen EBNA2 condensates in live cells are disrupted by 2% 1,6-hexanadiol (related to Fig.2e).

Movie 2. scFv-mNeonGreen EBNA2 condensates in live cells are disrupted by 5% 1,6-hexanadiol (related to Fig.2e).

Movie 3. scFv-mNeonGreen EBNA2 condensates in live cells are disrupted by 10% 1,6-hexanadiol (related to Fig.2e).
