# Supplementary material for: Phase separation of Epstein-Barr virus EBNA2 protein reorganizes chromatin topology for epigenetic regulation
Source: Commun Biol. 2021 Aug 16;4:967. doi: 10.1038/s42003-021-02501-7 (PMC8368186; doi:10.1038/s42003-021-02501-7)
Supplement: Supplementary file 2 — Description of Additional Supplementary Files [file 42003_2021_2501_MOESM2_ESM.pdf]

Description of additional supplementary items

Title: Supplementary Movie 1.

Description: scFv-mNeonGreen EBNA2 condensates in live cells are disrupted by 2% 1,6-hexanadiol (related to Fig.2e).

Title: Supplementary Movie 2.

Description: scFv-mNeonGreen EBNA2 condensates in live cells are disrupted by 5% 1,6-hexanadiol (related to Fig.2e).

Title: Supplementary Movie 3.

Description: scFv-mNeonGreen EBNA2 condensates in live cells are disrupted by 10% 243 1,6-hexanadiol (related to Fig.2e).

Title: Supplementary Data 1

Description: The source data underlying all figures
