# Supplementary material for: Phase separation of Epstein-Barr virus EBNA2 protein reorganizes chromatin topology for epigenetic regulation
Source: Commun Biol. 2021 Aug 16;4:967. doi: 10.1038/s42003-021-02501-7 (PMC8368186; doi:10.1038/s42003-021-02501-7)
Supplement: Supplementary file 7 — Reporting summary [file 42003_2021_2501_MOESM7_ESM.pdf]

nature research

Corresponding author(s): *Double-blind peer review submissions: write DBPR and your manuscript number here instead of author names.*

Last updated by author(s): *YYYY-MM-DD*

Reporting Summary

Nature Research wishes to improve the reproducibility of the work that we publish. This form provides structure for consistency and transparency in reporting. For further information on Nature Research policies, see our Editorial Policies and the Editorial Policy Checklist.

Statistics

For all statistical analyses, confirm that the following items are present in the figure legend, table legend, main text, or Methods section.

n/a	Confirmed	
☐	☒	The exact sample size (*n*) for each experimental group/condition, given as a discrete number and unit of measurement
☐	☒	A statement on whether measurements were taken from distinct samples or whether the same sample was measured repeatedly
☐	☒	The statistical test(s) used AND whether they are one- or two-sided<br>*Only common tests should be described solely by name; describe more complex techniques in the Methods section.*
☒	☐	A description of all covariates tested
☒	☐	A description of any assumptions or corrections, such as tests of normality and adjustment for multiple comparisons
☐	☒	A full description of the statistical parameters including central tendency (e.g. means) or other basic estimates (e.g. regression coefficient) AND variation (e.g. standard deviation) or associated estimates of uncertainty (e.g. confidence intervals)
☒	☐	For null hypothesis testing, the test statistic (e.g. *F*, *t*, *r*) with confidence intervals, effect sizes, degrees of freedom and *P* value noted<br>*Give P values as exact values whenever suitable.*
☒	☐	For Bayesian analysis, information on the choice of priors and Markov chain Monte Carlo settings
☒	☐	For hierarchical and complex designs, identification of the appropriate level for tests and full reporting of outcomes
☒	☐	Estimates of effect sizes (e.g. Cohen's *d*, Pearson's *r*), indicating how they were calculated

Our web collection on statistics for biologists contains articles on many of the points above.

Software and code

Policy information about availability of computer code

Data collection

Fluorescence images were obtained by Nikon NIS-Elements AR (v5.10.01) or ZEN (v2 or ZEN Blue).
Western blot images were obtained by Image Lab (v3.0).

Data analysis

Image processing: FIJI (v.1.52p), Nikon NIS-Elements Viewer (v4.20) .
IDR prediction: PONDR VSL2 (http://www.pondr.com/), IUPred (https://iupred2a.elte.hu/).
FRAP analysis: Nikon NIS-Elements AR (v5.10.01), Excel Office 365.
Colocalization analysis: ImageJ Plot Profile tool.
Foci counting and analysis: 3D Object Counter plugin in FIJI (https://imagej.nih.gov/ij/plugins/track/objects.html)
Hi-C Analysis
All Hi-C libraries were sequenced either on an Illumina HisseqXten-PE150. For each sample,reads were obtained following quality filtering and adaptor trimming using fastp (v0.20.0) with parameter '--thread 8 -5 -3 –W 4 '. Hi-C mapping, filtering, correction, and binning were performed with the HiC-Pro(v2.11.1) software(https://github.com/nservant/HiC-Pro). The paired-end reads were mapped to the UCSC human genome assembly(hg38). Singleton, multi-mapped, low-quality,unmapped,dumped, dangling, self-circle paired-end reads, and PCR duplicates were all removed by HiC-Pro after mapping.We generated raw contact matrices at 10-kb,20-kb,50-kb,100-kb,500-kb,1-Mb resolutions.For raw contact matrices correction from Hi-C data,we used the iterative correction method(ICE) through HiC-Pro software.The hicpro2juicebox.sh utility was used to convert the allValidPairs output of the pipeline into Juicebox .hic format at fragment resolution.Visualization of Hi-C contact matrices was done via juicerbox (https://github.com/aidenlab/juicer/wiki/Download).
Identification of chromatin loops
Chromatin loops in CNE-2 S18 cells control and CNE-2 S18 cells EBNA2+ cells were called using Fit-Hi-C(v2.0.7).First,input files of Fit-Hi-C were created by using a publicly available script(hicpro2fithic.py) from HiC-Pro.Next,for Fit-Hi-C, loops were called using fixed-size bin resolutions from 10 to 25 kb in both cell types.Briefly, significant interaction loops (q <= 0.05) were identified through jointly modeling the contact probability using raw contact frequencies and ICE normalization vectors with the Fit-Hi-C algorithm.
Meta-analysis of loops
The popular format to store Hi-C data, .hic, can be converted into .cool files using hic2cool(v0.8.3) software (https://github.com/4dn-dcic/

hic2cool). Hi-C matrices in cool format were used to generate genome-wide aggregate plots at loops of HEK293T cells control and HEK293T EBNA2+ cells detected by Hi-C. We used coolpup.py(v0.9.5) to pile-up normalized Hi-C signals at a 25-kb resolution at loops previously identified, and plotted 600-kb upstream and downstream of the loop anchor coordinates(https://github.com/open2c/coolpuppy). We ploted them using plotpup.py(v0.9.5).

ChIP-Seq Analysis

High-confidence reads of ChIP-Seq data obtained by using fastp(v 0.20.0) with parameter '--thread 8 -5 -3 –W 4 ', were mapped to human genome hg38 by using Bowtie2(v2.3.5.1) with parameters (--sensitive, –p 6), and PCR duplicated fragments were filtered by Picard(v2.22.8) (Picard Toolkit 2019, Broad Institute,GitHub Repository, https://broadinstitute.github.io/picard/).Then,we filtered the unmapped, multi-mapped reads and mapping to the reads on chrM. FRiP (fragments ratio in peaks) value was calculated by using bedtools(v2.29.2) and awk(v4.0.2). We used deepTools to generate bigWig file with RPKM normalization,and these files can be visualized in IGV. Peaks were identified by HOMER that contains a program called findPeaks with parameters (–style histone). Enriched peaks region generated by HOMER software was used as input to DESeq2(v1.30.0) to find differential peaks from ChIP-Seq data as well as normalised the data.

ATAC-Seq Analysis

Raw sequence reads were initially processed for removing adapter sequences and poor quality reads by Fastp(v 0.20.0). Subsequently ,the remaining reads were mapped to the human genome hg38 using Bowtie2(v2.3.5.1) with parameters （-- sensitive,-X 2000） . PCR duplicated fragments were filtered by Picard(v2.22.8). Then,we filtered the unmapped, multi-mapped reads and mapping to the reads on chrM. FRiP (fragments ratio in peaks) value was calculated by using bedtools(v2.29.2) and awk(v4.0.2). We used deepTools to generate bigWig file with RPKM normalization,and these files can be visualized in IGV. SAM files were converted to BAM format using Samtools and used for peak calling. MACS2(v2.2.4) with parameters(-t input_file –q 0.01 -f BAM --nomodel --shift -100 --extsize 200 -keep-dup all)was used to call peaks.

CUT&Tag Analysis

Fastp(v 0.20.0) with parameter '--thread 8 -5 -3 –W 4 ' was used to remove adapter and low-quality reads.Align paired-end reads used Bowtie2(v2.3.5.1) with the following parameters:-p 8, --sensitive.Duplicated reads were removed using Picard(v2.22.8) with this parameter: REMOVE_DUPLICATES = true.Then,we filtered the unmapped, multi-mapped reads and mapping to the reads on chrM. FRiP (fragments ratio in peaks) value was calculated by using bedtools(v2.29.2) and awk(v4.0.2). We used deepTools to generate bigWig file with RPKM normalization,and these files can be visualized in IGV. Peak calling used HOMER that contains a program called findPeaks with parameters (–style histone). Enriched peaks region generated by HOMER software was used as input to DESeq2(v1.30.0) to find differential peaks from CUT-Tag data as well as normalised the data.

Annotation of ChIP , ATAC and CUT-Tag peak sets

To obtain a peak set per condition, we first overlapped the peaks in each replicate and then only the peaks present in both replicates were considered.We used the ChIPSeeker library to annotate the peak sets obtained. Annotation packages: "TxDb.Hsapiens.UCSC.hg38.knownGene" and "org.Hs.eg.db" (Bioconductor). Promoters were defined as ± 3 kb from the transcription start site.Venn diagrams were generated using Intervene(v0.6.4).Heatmaps and average profiles were performed on bigWig files using deepTools(v3.5.0) plotHeatmap.

Motif Analysis

To analyze the enriched motifs in the peaks from ChIP-seq data,we used the findMotifsGenome.pl to identify enriched motifs from HOMER software.

For manuscripts utilizing custom algorithms or software that are central to the research but not yet described in published literature, software must be made available to editors and reviewers. We strongly encourage code deposition in a community repository (e.g. GitHub). See the Nature Research guidelines for submitting code & software for further information.

Data

Policy information about availability of data

All manuscripts must include a data availability statement. This statement should provide the following information, where applicable:

- Accession codes, unique identifiers, or web links for publicly available datasets
- A list of figures that have associated raw data
- A description of any restrictions on data availability

Next-generation sequencing data from ChIP-Seq, ATAC-Seq, CUT&Tag and Hi-C experiments for this current study are deposited in the NCBI GEO under accession number GSE158288.

Field-specific reporting

Please select the one below that is the best fit for your research. If you are not sure, read the appropriate sections before making your selection.

☒ Life sciences ☐ Behavioural & social sciences ☐ Ecological, evolutionary & environmental sciences

For a reference copy of the document with all sections, see nature.com/documents/nr-reporting-summary-flat.pdf

Life sciences study design

All studies must disclose on these points even when the disclosure is negative.

Sample size	No statistical approach was used to predetermine sample size. The cell counting, RT-qPCR, ATAC-see were conducted with three independent experiments. ChIP-seq of CNE-2 cells was conducted with two independent samples (for example, either scFv mNeonGreen or EBNA2 ChIP-seq). CUT&Tag of BJAB cells was conducted with two independent samples (for example, either scFv mNeonGreen or EBNA2 CUT&Tag). Hi-C was conducted with two independent experiments.
Data exclusions	No data were excluded.

Replication	All experiments were reproduced reliably in independent setting for at least two or three times.
Randomization	All experiments were reproduced reliably in independent setting for at least two or three times.
Blinding	No groups.

Reporting for specific materials, systems and methods

We require information from authors about some types of materials, experimental systems and methods used in many studies. Here, indicate whether each material, system or method listed is relevant to your study. If you are not sure if a list item applies to your research, read the appropriate section before selecting a response.

Materials & experimental systems

n/a	Involved in the study
☐	☒ Antibodies
☐	☒ Eukaryotic cell lines
☒	☐ Palaeontology and archaeology
☒	☐ Animals and other organisms
☒	☐ Human research participants
☒	☐ Clinical data
☒	☐ Dual use research of concern

Methods

n/a	Involved in the study
☐	☒ ChIP-seq
☒	☐ Flow cytometry
☒	☐ MRI-based neuroimaging

Antibodies

Antibodies used	anti-GAPDH (10494-1-AP, Proteintech, USA), anti-HA-Tag (C29F4, Cell Signaling Technology, USA), anti-Histone H3 (ab1791, Abcam, USA), anti-Histone H3 (acetyl K27) (ab4729, Abcam, USA), anti-FLAG (66008-3-1g, proteintech, USA), anti-p300 (ab54984, Abcam, USA), anti-rabbit IgG (FD0218, Fudebio, China), anti-mouse IgG DyLightTM 549 (610-442-002, ROCKLAND, USA), anti-DAPI (C1002, Beyotime, China)
Validation	anti-FLAG (66008-3-1g, proteintech) validated by detection of EBNA2 truncated single band at predicted molecular weight which was transfected into HEK293T cells. anti-GAPDH (10494-1-AP, Proteintech), anti-Histone H3 (ab1791, Abcam) and anti-HA-Tag (C29F4, Cell Signaling Technology) were validated by Western blotting. anti-mouse IgG DyLightTM 549 (610-442-002, ROCKLAND), anti-p300 (ab54984, Abcam) and anti-DAPI (C1002, Beyotime) were validated by immunofluorescence or ATAC-see. anti-Histone H3 (acetyl K27) (ab4729, Abcam) was validated by Western blotting, immunofluorescence, ChIP-seq and CUT&Tag. anti-rabbit IgG (FD0218, Fudebio) was validated by CUT&Tag.

Eukaryotic cell lines

Policy information about cell lines

Cell line source(s)	Cell lines used in the study included HEK293T (ATCC #CRL-3216), CNE-2 (RRID #CVCL_6889), BJAB (RRID #CVCL_5711).
Authentication	Cell lines were authenticated by short tandem repeat (STR) analysis. Growth rates and cell morphology of the cell lines used in this study were similar to previous reports.
Mycoplasma contamination	Cell lines were routinely tested for mycoplasma contamination by PCR and DAPI/Hoescht staining. All tests were negative.
Commonly misidentified lines (See ICLAC register)	No commonly misidentified cell lines were used in the study.

ChIP-seq

Data deposition

☒ Confirm that both raw and final processed data have been deposited in a public database such as GEO.

☒ Confirm that you have deposited or provided access to graph files (e.g. BED files) for the called peaks.

Data access links *May remain private before publication.*	https://www.ncbi.nlm.nih.gov/geo/query/acc.cgi?acc=GSE158288
Files in database submission	S18 Dox10ug input rep1<br>S18 Dox10ug input rep2<br>S18 Dox10ug-H3K27ac rep1<br>S18 Dox10ug H3K27ac rep2<br>S18 EBNA2Con input Rep1<br>S18 EBNA2Con input Rep2

S18 EBNA2Con H3K27ac rep1
S18 EBNA2Con H3K27ac rep2
S18 EBNA2Dox10ug input rep1
S18 EBNA2Dox10ug input rep2
S18 EBNA2Dox10ug H3K27ac rep1
S18 EBNA2Dox10ug H3K27ac rep2

Genome browser session (e.g. UCSC)

no longer applicable

Methodology

Replicates

We have used three different tags(Dox10ug,EBNA2Con,EBNA2Dox10ug).

Sequencing depth

sample name raw reads mapped reads Read type Read length
S18 Dox10ug input rep1 11500993 10770452 paired-end 150
S18 Dox10ug input rep2 18061151 16664710 paired-end 150
S18 Dox10ug-H3K27ac rep1 17085290 15278251 paired-end 150
S18 Dox10ug H3K27ac rep2 14053330 13591702 paired-end 150
S18 EBNA2Con input Rep1 18794419 17203404 paired-end 150
S18 EBNA2Con input Rep2 17500270 16959249 paired-end 150
S18 EBNA2Con H3K27ac rep1 13419689 11804891 paired-end 150
S18 EBNA2Con H3K27ac rep2 17143673 16685905 paired-end 150
S18 EBNA2Dox10ug input rep1 13001451 11621896 paired-end 150
S18 EBNA2Dox10ug input rep2 18136069 17649221 paired-end 150
S18 EBNA2Dox10ug H3K27ac rep1 15514412 13989265 paired-end 150
S18 EBNA2Dox10ug H3K27ac rep2 15050977 14503141 paired-end 150

Antibodies

anti-Histone H3 (acetyl K27) (ab4729, Abcam, USA)

Peak calling parameters

HOMER that contains a program called findPeaks with parameters:–style histone

Data quality

fastp(v 0.20.0)

Software

fastp(v 0.20.0),Bowtie2(v2.3.5.1),Picard(v2.22.8),bedtools(v2.29.2),awk(v4.0.2),deepTools(v3.5.0),HOMER (version 4.10.4)
